# Module Discovery by Exhaustive Search for Densely Connected, Co-Expressed Regions in Biomolecular Interaction Networks

**DOI:** 10.1371/journal.pone.0013348

**Published:** 2010-10-25

**Authors:** Recep Colak, Flavia Moser, Jeffrey Shih-Chieh Chu, Alexander Schönhuth, Nansheng Chen, Martin Ester

**Affiliations:** 1 School of Computing Science, Simon Fraser University, Burnaby, Canada; 2 Center for Disease Control, University of British Columbia, Burnaby, Canada; 3 Department of Molecular Biology and Biochemistry, Simon Fraser University, Burnaby, Canada; 4 Department of Mathematics, University of California, Berkeley, California, United States of America; Memorial Sloan-Kettering Cancer Center, United States of America

## Abstract

**Background:**

Computational prediction of functionally related groups of genes (*functional modules*) from large-scale data is an important issue in computational biology. Gene expression experiments and interaction networks are well studied large-scale data sources, available for many not yet exhaustively annotated organisms. It has been well established, when analyzing these two data sources jointly, modules are often reflected by highly interconnected (*dense*) regions in the interaction networks whose participating genes are co-expressed. However, the tractability of the problem had remained unclear and methods by which to exhaustively search for such constellations had not been presented.

**Methodology/Principal Findings:**

We provide an algorithmic framework, referred to as *Densely Connected Biclustering (DECOB)*, by which the aforementioned search problem becomes tractable. To benchmark the predictive power inherent to the approach, we computed all co-expressed, dense regions in physical protein and genetic interaction networks from human and yeast. An automatized filtering procedure reduces our output which results in smaller collections of modules, comparable to state-of-the-art approaches. Our results performed favorably in a fair benchmarking competition which adheres to standard criteria. We demonstrate the usefulness of an exhaustive module search, by using the unreduced output to more quickly perform *GO term related* function prediction tasks. We point out the advantages of our exhaustive output by predicting functional relationships using two examples.

**Conclusion/Significance:**

We demonstrate that the computation of all densely connected and co-expressed regions in interaction networks is an approach to module discovery of considerable value. Beyond confirming the well settled hypothesis that such co-expressed, densely connected interaction network regions reflect functional modules, we open up novel computational ways to comprehensively analyze the modular organization of an organism based on prevalent and largely available large-scale datasets.

**Availability:**

Software and data sets are available at http://www.sfu.ca/~ester/software/DECOB.zip.

## Introduction

On the cellular level, life is driven by chemical compounds acting in concert, in response to internal and external signals. The ultimate goal of investigating the underlying complex molecular patterns is to draw detailed maps of cellular mechanisms, such as metabolic pathways, and their interplay. However, the challenges behind a comprehensive computational and experimental exploration of these mechanisms seem to be daunting, due to the tremendous complexity of living organisms.

The modularity paradigm [1] provides a key insight how to computationally overcome the inherent difficulties in a first important step. This paradigm states that functional subunits of the cellular maps are encoded as *modules*, i.e. groups of functionally related genes. As a most relevant example of practical interest, knowledge about a module facilitates to assign functions to not yet annotated genes modularly associated with fully annotated functional “collaborators”. Therefore, the design of biologically sound as well as computationally tractable models for inferring modules has been at the core of functional genomics throughout the post-genomic era.

When searching for modules, approaches that integrate several types of data promise to be superior. Well-known general aspects which support combined analyses are increased robustness with respect to the ubiquitous noise in large-scale data, the global correlation between the ‘*omic*’ data types [2], [3] and that single data types provide only partial information. In particular, when jointly analyzing gene expression and interaction data one should consider that:

- Many cellular processes cannot be monitored by studying gene expression alone. For example, several cell-cycle related protein complexes in Yeast contain predominantly housekeeping gene products such that the functional coherence of the genes of these complexes is not visible on the transcriptional level [4]. However, when defined appropriately, co-expressed groups of genes tend to reliably reflect functional modules.
- Subgroups of genes, inferred by screening interaction network data, exhibit quite the opposite behavior. While many more cellular functionalities are reflected by connected subnetworks, the likelihood that a connected subnetwork reflects a functional module is comparatively low. This is due to the fact that interaction networks provide only static pictures of the cell such that the edges in a connected subgraph might not be simultaneously present.
- Based on these two insights we hypothesized that to combine a rather strict network metric (here: *dense* connectivity) with a more relaxed gene expression metric (our definition of co-expression is little restrictive) may result in an excellent, while at the same time computationally manageable definition of a functional module.

In the meantime, a variety of reliable and sound approaches to module discovery has been provided to the related communities. However, some open questions had remained. In particular, none of the established approaches which integrate both network and gene expression data fully resolves the following issues. (There are non-integrated approaches which address the issues from below.)

- They only provide heuristic solutions to the biologically well-motivated (e.g. [5]–[9]), albeit computationally hard problem of searching for densely connected biclusters (in the sense of densely interconnected regions in interaction networks whose participating genes are co-expressed under sufficiently many cellular conditions). Note that *density*, in addition to connectivity, is a recurring theme in approaches based on network data alone (see [10] for a summary).
- They tend to partition the datasets. However, overlap among the modules is desirable since genes can participate in several, sometimes substantially different, functional contexts.
- While large collections of modules are usually of no immediate practical use, they can be flexibly transformed into smaller outputs of particular interest since they cover the maximum amount of functionalities that can be inferred from the underlying datasets. None of the existing approaches outlines such strategies since they do not find large collections in the first place.
- Gene expression modules tend to reliably reflect functional modules in terms of GO term enrichment, but they do not cover many functionalities since many functionalities do not show on the mRNA level. Network modules show the opposite effect—they achieve good coverage of functionalities since an interaction network usually covers all genes independent of tissue, condition etc. However, network modules often are false positives precisely due to that one cannot ensure that two interactions are active under the same conditions. Combined approaches aim at yielding balanced combinations of enrichment and coverage. However, approaches yielding both enrichment which is on par with methods based on gene expression data alone and coverage comparable with approaches based on network data alone had not been presented yet.

### Approach

**Figure 1:**
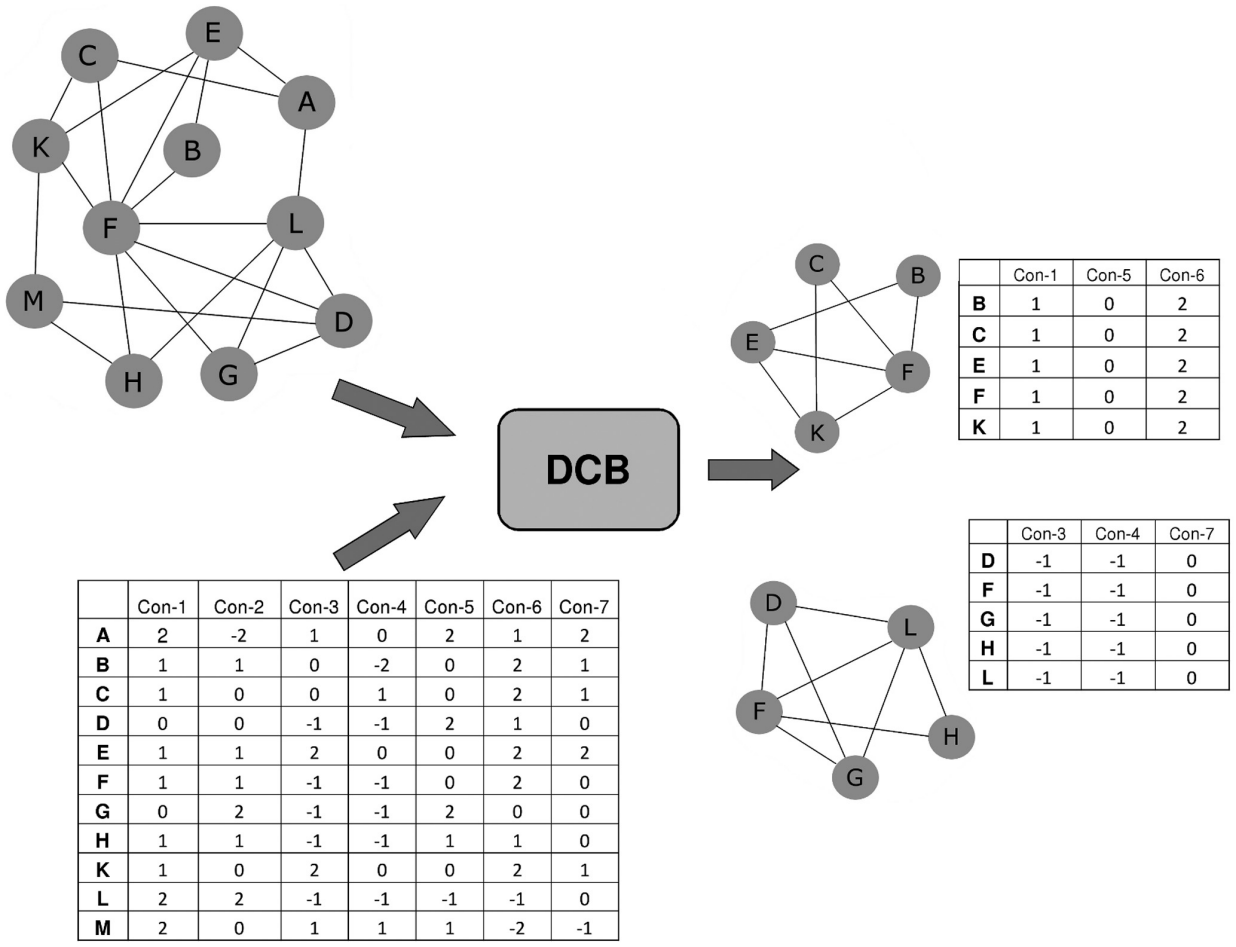
This figure refers to the definition of a densely connected bicluster (see Methods section, definition 1) referring to the parameters 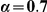 (density) and 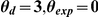 (co-expression constraints). The input for the core algorithm is the interaction network of the organism (here, as a toy example, genes A,B,C,D,E,F,G,H,I,J and K) together with gene expression dataset containing (logarithmic) fold changes of genes across a set of experimental conditions (here: the table below the interaction network). On the right, we display the set of densely connected biclusters which refer to the datasets on the left. The densely connected biclusters contain at least 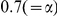 times the amount of possible edges and its genes are co-expressed in at least 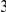 different experimental conditions (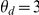) with a difference of at most 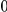 (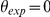).

The major purpose of this study was to outline ways to exhaustively search for densely connected biclusters in biomolecular network and gene expression data and to elucidate the advantages of such an approach in the light of the four points from above. We do this by employing a search strategy which was recently presented to the data mining community [11] and tayloring it to the particular requirements when performing functional module discovery. As an illustration see Figure 1. An exhaustive search for maximal densely connected biclusters among the genes 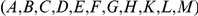 results in two subgroups: 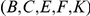 and 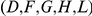 both of which are connected and contain at least 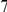 of the possible 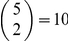 edges in the interaction subnetwork (which translates to density 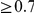). These two groups of genes also form biclusters since all of the genes are co-expressed in at least 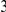 conditions (Con-1, Con-5, Con-6 for 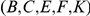 and Con-3, Con-4, Con-7 for 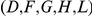). See the Methods section for a formal introduction of the related theory.

The basic idea behind the strategy is to examine all subnetworks of the interaction network for forming a densely connected bicluster but those which can be neglected based on a theorem which was presented in [11]. Hereby, the theoretical advance is to observe that this renders the computational search problem tractable when screening interaction in combination with gene expression data. The search proceeds in a breadth-first fashion which translates to first screening all subnetworks of size 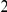 and proceeding with subnetworks of size 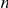 when having enumerated those of size 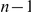. Based on the theorem from [11], we can neglect subnetworks of size 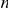 whenever all subnetworks of size 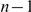 contained in the subnetwork of size 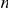 are not densely connected biclusters. The theorem ensures that we will not miss a densely connected bicluster. See the Results section, subsection “Tractability: Runtime Analysis” for a runtime analysis which shows that our method has reasonable runtimes on the real-world instances considered.

After subsequent application of a novel merging and a novel statistical ranking procedure, we obtain a collection of modules of great quality where modules possibly overlap. The quality of our modules is documented by performing highly favorably in a benchmarking competition. Most importantly, our approach is the only one to achieve top-ranked enrichment and top-ranked coverage simultaneously. Furthermore, we can demonstrate that the overlap among the modules can help to discover different functions of the same gene supported by that a gene may participate in different modules reflecting different functionalities. We also show that the comprehensiveness of our output can be used to perform *function specific* module discovery which will be addressed in the Results section, subsection “”Advantage of Exhaustive Searches”.

### Related Work

#### Non-Integrative Approaches

In large-scale gene expression data, a module is usually defined as a group of co-expressed genes. Several approaches have demonstrated that co-expression significantly increases the likelihood for a group of genes to have similar function (see e.g. [12]–[14] for seminal papers). Recently, a large variety of inference and clustering algorithms have been presented, often specializing in more specific problem domains. A class of methods that is related to ours are biclustering algorithms. Since the definition of a bicluster is that of a cluster of both genes and cellular conditions, this class of algorithms is particularly suitable when it comes to simultaneously analyzing gene expression data resulting from experiments referring to various different cellular conditions [15], [16]. Here, *SAMBA* [17] proved to be a superior approach in a recent comparative study [18].

Network-based methods for function prediction have been comprehensively reviewed [10]. Various network-clustering algorithms and related approaches have been presented since the availability of large-scale network data (e.g. [19]–[22], see also the citations in [10]). In an independent comparative study [23], *MCL*, a Markov chain based method [24], [25] performed most favorably on the suggested benchmarking datasets. Apart from the fact that modules are reflected by connected subgraphs in interaction networks, it is well-established that they usually are also *dense* in terms of above-average edge content. This applies in particular for protein-protein interaction networks (e.g. [8], [9]) since the physical interaction of two gene products is vital for the two genes to commonly exert function. Note that [9] is the only approach which tries to exhaustively mine for densely connected subnetworks. However, they can only prove to find all dense, but not necessarily connected subnetworks. As a consequence, the devised search strategy can provably miss certain densely connected constellations. Moreover, they do not address how to integrate gene expression data.

#### Integrated Approaches

A recurring theme in earlier approaches is to infer modules as connected subnetworks where genes are co-expressed. In the two seminal approaches, Ideker et al. [26] find connected subnetworks which yield a high score measured in P-values obtained from gene expression experiments whereas Hanisch et al. [27] define distance functions, based on both expression and network information, which are subsequently integrated into standard clustering procedures. Segal et al. [28] provided probabilistic graphical models with which to perform combined analysis of interaction network and gene expression data, thereby establishing the first unifying statistical approach to the issue.

Recently, integrated methods aim at inferring modules as *densely connected* regions in interaction networks that is regions which are not only connected but also contain a high amount of edges, certainly inspired by the successes of approaches based on network data alone which made use of this idea. In fact, it is well-established that when combining interaction network and gene expression data, modules are often reflected by *densely connected biclusters*, that is, dense and connected regions in the interaction networks where the participating genes are co-expressed under a sufficient number of cellular conditions [8]. However, the tractability of the corresponding computational search problem had never been demonstrated and all of the previous approaches present related heuristics.

In the most recent approach, Ulitsky and Shamir [29] compute connected subnetworks which, according to a statistical hypothesis test, are significantly co-expressed. Ulitsky and Shamir [29] also report that they outperform state-of-the-art approaches in terms of GO term enrichment and coverage (for definitions see Results section, subsection “Module Assessment”). The modules inferred in [29] are relatively dense (see Table 1 and 2) which can be taken as an additional indicator of that functional modules are associated with densely connected, co-expressed subnetworks. Note as well that [29] employ a heuristic, the “heaviest-subnet algorithm” which computes densely connected interaction subnetworks to be used as seeds in the subsequent main algorithm. However, their method does not solve the problem of exhaustively searching all such subnetwork patterns. See the supplementary materials File S1 for a more detailed description. Note also that there are recent approaches addressing how to reliably make use of confidence-scored interaction networks (e.g. [30]). In the following, we do not compare with such methods since confidence scores require a substantial amount of annotations to be trustworthy. Therefore, such approaches refer to a different, though related, problem domain. Here, we would like to focus on module discovery approaches which do not intrinsically rely on annotations.

**Table 1:** Benchmarking competition yeast.

	Basic Statistics				Quality Measures		
Benchmarking Competitors	#Gen.	#Mod.	AMS	DY	ER	COV	IC
*SAMBA*	876	135	25.96	.02	90 (**2**)	11	20
*MCL*	693	95	7.29	.44	88	30 (**1**)	33 (**2**)
*Matisse*	360	17	21.17	.31	95 (**1**)	6	17
*COC*	986	103	9.57	.06	72	19	16
*Rand. Conn.*	737	134	16.87	.27	84	23	4
*DECOBRA*	576	354	9.33	.41	95 (**1**)	29 (**2**)	41 (**1**)

Additional Methods	#Gen.	#Mod.	AMS	DY	ER	COV	IC
*DECOBRA* Top-100	226	100	13.14	.33	100	5	18
*DECOBRA* Top-200	388	200	11.49	.35	97	16	31
*DECOB*	576	2276	9.33	.39	93	46	55

**Table 2:** Benchmarking competition human.

	Basic Statistics				Quality Measures		
Benchmarking Competitors	#Gen.	#Mod.	AMS	DY	ER	COV	IC
*SAMBA*	1709	129	48.94	.01	95 (**1**)	13	12
*MCL*	1863	312	5.94	.35	81	58	27 (**2**)
*Matisse*	1364	76	17.94	.30	93 (**2**)	25	18
*COC*	3558	271	13.12	.01	79	44	7
*Rand. Conn.*	1921	406	10.18	.35	88	61 (**1**)	3
*DECOBRA*	1358	758	6.52	.46	95 (**1**)	60 (**2**)	37 (**1**)

Additional Methods	#Gen.	#Mod.	AMS	DY	ER	COV	IC
*DECOBRA* Top-100	347	100	7.12	.44	97	15	10
*DECOBRA* Top-200	553	200	7.08	.44	95	26	15
*DECOB*	1358	5979	7.12	.45	97	64	51

#### Interaction Data

Beyond being applicable for physical interaction networks, the definition of dense connectivity also makes sense when screening genetic interaction networks for modules [5]. While the correlation of genetic interaction subnetwork patterns with functional entities has not yet been fully explained, a densely connected region in a genetic interaction network usually gives rise to a module. Note, however, that there are exceptions, such as bridge genetic interactions that exist between pathways as compared to within pathway interactions [7]. These cases do not necessarily form a dense region in a genetic interaction network. In summary, finding densely connected regions in genetic interaction networks alone should yield that the modules are quite trustworthy while not necessarily all modules are discovered. Note also that genetic interaction data and physical protein-protein interaction data are often complementary [7]. For example, this was made use of for understanding gene interaction modules in C. elegans early embryogenesis [6] as well as LIN-12-Notch signalling and the actin cytoskeleton pathways [31]. Therefore, combining those two data types can be advantageous.

## Results

First, we computed all densely connected biclusters in both Yeast and Human according to definition 1. We then distinguish between two methods which result from further processing the exhaustive set of all these densely connected biclusters. The output of the first, called *DECOB (DEnsely COnnected Biclustering)* is obtained by subsequently merging biclusters which share a large dense core. This is motivated by that biclusters which substantially overlap do not differ much in terms of their functional interpretation. See also [32] for a related discussion. We refer to the set of biclusters where substantially overlapping modules heve been merged as *DECOB* modules in the following.

The output of the second method, *DECOBRA (DECOB RAnked)*, has been specifically tailored to serve the purposes of a fair benchmarking procedure. It consists of the *DECOB* modules which remain after having applied an automatized ranking-based filtering procedure to the *DECOB* modules which results in a reduced number of modules, referred to as *DECOBRA* modules. See the Methods section, subsection “DECOBRA: Algorithm” for a full description of the ranking-based filtering procedure.

We computed all densely connected biclusters in both Human and Yeast, based on standard gene expression and protein-protein / genetic interaction network datasets (see the Methods section, subsection “Data” for a more detailed explanation). In order to demonstrate the benefits of our approach we then computed all *DECOB* modules and, by means of the above mentioned filtering procedure, the *DECOBRA* modules. We then

1. performed a standard benchmarking competition (see subsection “Standard Function Predictionn Benchmarking” below) for which we suggest *DECOBRA* as a fair competitor and
2. evaluated the (unreduced) set of *DECOB* modules when employed for specific function prediction tasks (see section “Advantage of Exhaustive Searches”) which require large and comprehensive sets of high-quality modules as a basis.

### Tractability: Runtime Analysis

**Figure 2:**
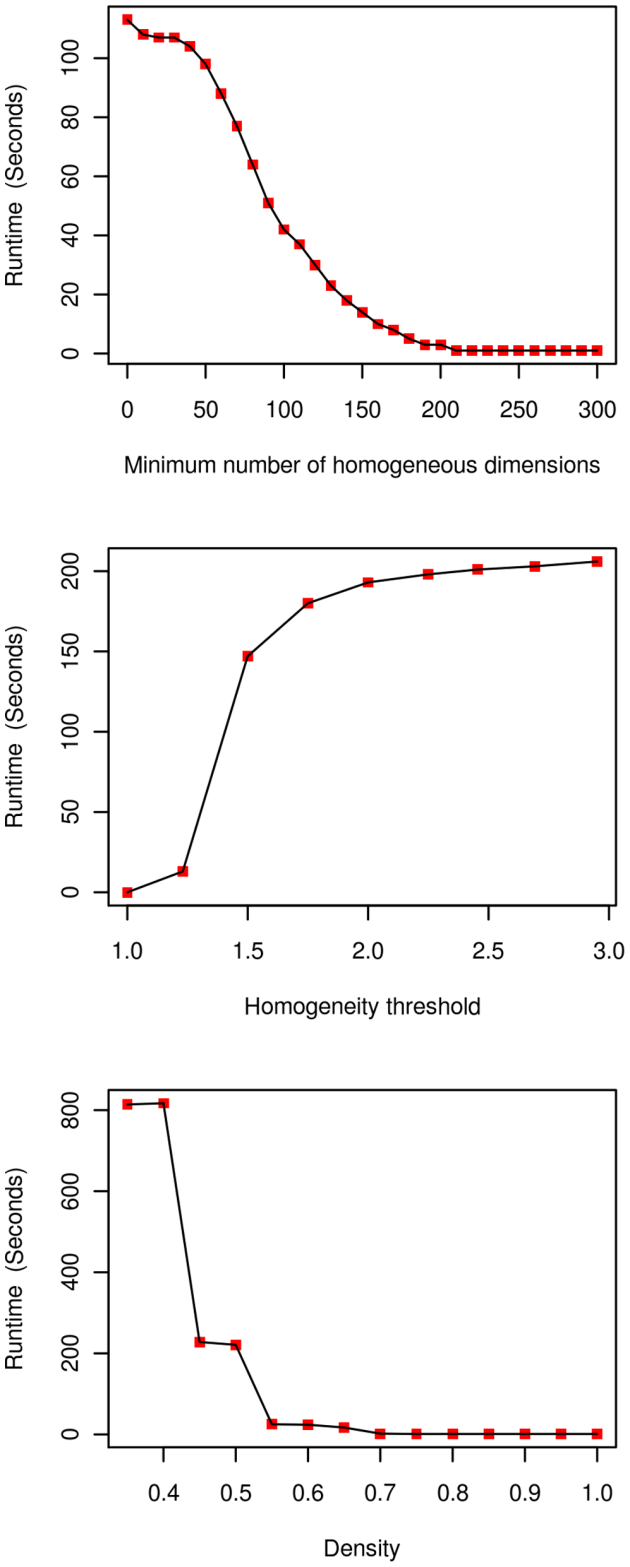
Runtimes of our algorithm for varying, biologically relevant choices of the parameters involved in our framework. The most important observation is that we have runtimes of at most a few minutes for all choices of 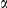 (density).

In order to give evidence that our approach achieves reasonable runtimes on biological problem instances of interest we tested our software on the Yeast dataset for varying choices of 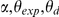 (for exact definitions of those parameters which quantify subnetwork density and co-expression, see the Methods section). Thereby, we left two of the parameters 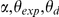 fixed at 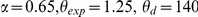 according to what was found a biologically motivated choice in Yeast and varied the third, remaining parameter. See Figure 2 for corresponding statistics. As one can see the combination of 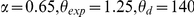 resulted in about 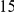 seconds runtime to process the Yeast dataset. Changing 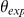 and 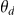 resulted in changes in runtime on the order of (up to 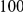) seconds (Figure 2, top and middle). Changing 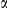 makes the most significant effects as was to be expected due to the exponential increase in search space size (Figure 2, bottom). As mentioned above, 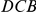 constraints are only loose anti-monotone for 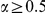 which requires to invoke additional subroutines in order to find all densely connected biclusters for choices of 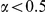 (see [11], [33] for details). However, even for the most problematic choices of 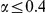 the runtime is only on the order of a few minutes beyond that such choices are biologically not necessarily well motivated in module discovery.

### Standard Function Prediction Benchmarking

The general outline of the following competition has been adopted from existing studies [29]. In the following, we refer to a group of potentially functionally related genes as inferred by any of the methodologies under consideration as a *(functional) module*. To directly compare the predictive power of the complete output of *DECOB* with those of the benchmarking competitors would be inappropriate since the complete output of *DECOB* is one order of magnitude larger than the outputs of the other methods in terms of inferred modules. The idea behind approaches yielding rather small outputs is to provide the experimenter with only a small collection of modules of utmost quality. Since the technologies behind the approaches of the competitors exclusively address this idea, a direct comparison of our collection with theirs would be misleading. Therefore, we developed a ranking procedure, which, when applied to the output of *DECOB* yields a result set that can be compared with the ones of the existing methods in a fair comparison. As mentioned above, we call the combined application of *DECOB* and the ranking-based filtering *DECOBRA*. procedure which yields the sort of output which can be incorporated into a meaningful benchmarking procedure as *DECOBRA*. In general, the output of *DECOBRA* can be used for common function prediction tasks in the sense of the earlier approaches.

As benchmarking competitors, we chose four related publicly available, state-of-the-art algorithms as well as a baseline method. The two integrated methods are *CO-Clustering (COC)* which is a seminal approach on the topic [27] and *MATISSE* [29] set the current standards. We also benchmarked against two methods that operate on single data types (either interaction network or gene expression data). While *MCL* [24] operates only on interaction network data, *SAMBA* [17] operates only on gene expression data (note that *SAMBA* can in theory also be used to integrate other types of data, but has not been thoroughly evaluated for such tasks. Both methods established the gold standard on the types of data under consideration. The baseline method (*Rand. Conn.*) randomly sampled connected PPI networks (we obtained empirical module size distributions from the output sets of all algorithmic approaches and sampled connected networks according to that size distribution). In the File S1 we provide a more detailed description of the algorithmic technologies which underlie the approaches of the competitors. Thereby, we put particular emphasis on the issues under special consideration here, such as overlap and density. For all algorithms, we used the recommended parameter settings if applicable.

### Module Assessment

We measured several GO-based quantities to assess module quality. The most important definitions of quantities have been adopted from earlier studies [29]. For all calculations, we used the high-throughput version of the GoMiner tool [34].

*Basic Statistics (# genes, # modules, average module size (AMS), Density (DY))*. These numbers provide insights about the number of genes covered by the inferred modules as well as the number of modules, their average size and their average density. These basic statistics may also assist in choosing convenient methods according to practical considerations. Average density (see Def. 1) reveals how density is related to module quality.

*Enrichment (ER)* is a standard measure and possibly the most important one. It can be interpreted as the probability that an inferred module is a set of functionally related genes. It is computed as the percentage of modules that are enriched with at least one GO term of level 7 or higher (meaning 8,9,…), as suggested in [29] with P-values corrected for multiple hypothesis testing, below a threshold of 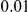. In this context, level means the length of the shortest directed path from the node associated with the most generic GO term to the target GO term based on the child-parent relations as induced by the topological organization of GO.

*Coverage (COV)* is another standard measure (see [29]). It is the number of GO terms that were enriched in any of the inferred modules divided by the number of all GO terms associated with the interaction network and gene expression datasets under consideration.

Lastly, as genes can be associated with GO terms reflecting different functionalities which indicates their participation in several functional contexts, we suggest *Individual Coverage (IC)* as a quantity which measures how well the functionalities of the individual genes are covered. IC is the probability that, given a gene and one of its associated GO terms, the GO term is enriched in one of the inferred modules containing that gene. More formally, if 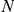 is the number of genes, let 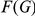 be the number of terms associated with gene 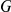 that are enriched in inferred modules that contain the gene and 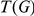 be the total number of terms associated with that gene then
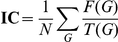
where the sum ranges over all genes 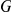. It measures how many of the functional contexts of a gene are covered by the output. In other words, it measures how well a method can identify multiple functions of a gene. Therefore, methods which yield non-overlapping modules have rather low IC (see Table 1 and 2).

In general, it is quite hard to provide a truly fair benchmarking competition, due to different numbers of covered genes and modules of the competitors. We suggest *DECOBRA* as a competitor since the number of covered genes is roughly the same as the one of the other methods. The output of *DECOBRA* results from application of a ranking based filtering procedure to the complete set of densely, connected and co-expressed interaction subnetworks (*DECOB* modules). Recall that the set of *DECOB* modules, without subsequent application of the ranking procedure results in substantially larger number of genes and modules (see the *DECOB* row in Table 1 and 2). We would finally like to point out that the design of strategies for comparison of clustering / module discovery methods which yield overlapping outputs is an active area of research (e.g. [23]). As mentioned above, we opted to have each method roughly the same amount of genes covered which is fair with respect to have everyone a “best bet” on the functions of the same amount of genes. The subsequent results on yeast and human dataset can generally be interpreted as the difference of having less, but usually larger, non-overlapping modules (competitors) in contrast to our approach which yields more, but smaller and overlapping modules.

#### Yeast

Table 1 displays the statistics, as defined in the Results section, subsection “Module Assessment”, that were achieved by the comparison partners on both yeast and human datasets (see Methods section, subsection “Data”). In each column of the table, the methods that perform best or second best (position in parentheses) are highlighted.

*DECOBRA* is first in ER and IC and second in COV. *MCL* wins COV, one obvious reason being that it assigns each gene to a module, thereby achieving high coverage rates. However, *MCL*'s performs rather poorly (relative to the baseline established by *Rand. Conn.*) in ER, which is considered to be the measure of individual module quality. This is likely due to yielding subnetworks as modules where edges are not simultaneously present since it does not consider gene expression data and confirms the intuitive idea about the limitations of static network data when it comes to function prediction. Nevertheless, recall that *MCL* proved to perform very favorably among the methods that consider network data alone [23] in an independent comparative study [23]. At any rate, it is interesting to observe the high density of the modules inferred by *MCL*. We would also like to mention the high enrichment value of *Matisse*. The relatively high density of the output modules (although this is not explicitly part of its underlying module definition) might come as no surprise. Clearly, a general explanation for *Matisse*'s module quality is that it is an integrated approach. Note that the only method which achieves both top-ranked enrichment as well as top-ranked coverage is *DECOBRA*.

#### Human

Table 2 displays the statistics defined in the Results section, subsection “Module Assessment”, that were achieved by the comparison partners on the human datasets (see Methods section, subsection “Data”). *DECOBRA* finishes shared first in ER, second in COV and first in IC. The baseline method *Rand. Conn.* wins COV. This points out that to use COV as the only quantity to measure coverage of function for a module discovery program is questionable. More appropriate quality measures are required. Note, for example, that *Rand. Conn.* performs suboptimal, if not poorly in the non-standard measures IC which we had suggested for further evaluation since they convey meaning of obvious interest in function prediction. In this context, note also that *MCL* performs slightly worse in COV, but superior (being second best) in IC. This further supports that coverage of functionalities is hard to assess and that novel ways for doing so are needed.

The high ER achieved by *SAMBA* is remarkable (sharing the first position with *DECOBRA*) which confirms that biclustering is a highly valuable approach when considering gene expression data alone. Moreover, it confirms that co-expression, if appropriately defined, is a strong indicator of functional relationships. However, *SAMBA*'s COV and IC are rather poor reflecting that not every functional relationship becomes visible in terms of co-expression *SAMBA* modules. *DECOBRA* employs a rather relaxed definition of co-expression whose predictive power comes from combining it with the retrieval of interaction relationships. Both *MCL* and *COC* achieve relatively good coverage values, again an obvious reason being that they assign each gene to a module. However, for both of them, ER is even worse than that of the baseline method (*Rand. Conn.* ). Last, note that *Matisse* achieves top-rated ER values also on the human dataset. In conclusion, note that *DECOBRA* is the only method to achieve both top-ranked ER and COV among all competitors.

### DECOB

In Table 1 and 2 we display results for the full set of *DECOB* modules. We also show the results for the top 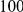 and top 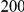 *DECOB* modules that result from stopping the filtering procedure after having filtered out 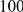 resp. 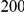 *DECOB* modules (*DECOBRA Top-100* resp. *DECOBRA Top-200*). *DECOB*'s high ER, in particular in Human (ER = 98) is quite remarkable since it can be related to that, in Human, 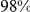 of all densely connected, co-expressed subnetworks are GO term enriched which underscores the applicability of the widely believed idea that such constellations reflect cellular functional entities. Furthermore, all of the top 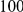 *DECOB* modules in Yeast are enriched. Last, note that *DECOB* achieves overall best values in COV and IC in both Human and Yeast. In accordance with the definitions of COV and IC, these demonstrates the benefits of *DECOB* when performing more specific function prediction tasks where large amounts of high quality modules are needed as a ground set. This will be described in subsection “Advantage of Exhaustive Searches” below.

### Advantages of Overlapping Modules

The benefits of allowing for overlap among modules are documented by the good IC values of *DECOBRA* (recall the definition of IC as the probability that a gene / GO term combination is reflected by a module containing the gene and being enriched relative to the GO term), in both Yeast and Human. Overlapping modules reflect different functional contexts where genes being part of the overlap play a role in all contexts affected. Note that the unreduced output of *DECOB* achieves even better IC values which gives evidence of the benefits of an exhaustive approach in this respect.

In Table 3 we have further evaluated how well the individual methods perform with respect to revelation of the different functionalities of the genes. It is obvious that overlap is a crucial necessity to properly reflect the different functionalities of a single gene. To further examine this we have counted all module pairs 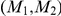 (OMPSDF = Overlapping Module Pairs Supporting Different Functionalities) in Table 3 such that

1. The intersection of 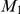 and 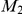 is not empty.

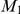
 and 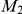 do not share (in terms of enrichment) a GO term at level 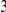. This translates to that they reflect different cellular core processes.
3. Among the genes which are shared by 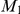 and 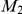 there is a gene which is annotated with two GO terms of level 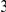 or below (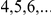) where one of the terms is enriched in 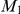 (hence not in 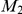, since 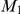 and 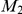 do not share such terms) and the other term is enriched in 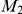 (hence not in 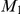). This means that the gene supports two functionalities which are essentially different.

**Table 3:** Statistics on overlapping module pairs supporting different functionalities (OMPSDF).

Organism	*SAMBA*	*MCL*	*Matisse*	*COC*	*DECOBRA*
Yeast	4	0	0	0	1264
Human	208	0	0	0	194

The number of such pairs of modules produced by the different methods are shown in Table 3. As expected, none of the methods which partition the datasets (*MCL*, *Matisse*, *COC*), in particular none of the existing combined ones (*Matisse*, *COC*) infers such module pairs. The only method apart from *DECOBRA* which outputs such configurations is *SAMBA*, which operates on gene expression data alone. The differences between the numbers in Yeast and Human are due to the peculiarities of the gene expression datasets under consideration. Note that the 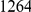 pairs reported correspond to 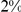 of the 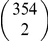 possible pairs where 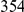 is the number of modules output by *DECOBRA* (see Table 1) which means that in Yeast 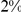 of the *DECOBRA* module pairs support the desirable idea of finding constellations where genes interact in different cellular functional contexts. Only *DECOBRA* reports substantial amounts of such overlapping configurations.

### Advantage of Exhaustive Searches

In the following, we demonstrate the advantages of an exhaustive module search by describing an experimental scenario of practical interest. The idea is to provide one or several functionalities of specific interest and then to select all modules from the output of a module discovery method which are enriched with functionalities under consideration. This aims at integrating partial knowledge in terms of functionalities in order to more specifically predict gene and protein function in rather sparsely annotated organisms. The resulting collections of modules should reflect functionalities which are related to the functionality specified. Since it is desirable to be able to add or combine functionalities interactively and not to have to recompute module collections upon modification of specification of functionalities, an advantageous workflow of such studies would be to

1. first compute a large collection of high quality modules and
2. then to interactively select collections of specific interest by simple filtering procedures.

Clearly, in order to support such an advantageous workflow, the initial collection should be both comprehensive and rich in terms of functionalities covered and reliable in terms of module quality. Revisiting the statistics of Table 1 and 2 reveals that the exhaustive collection of *DECOB* modules meets these criteria since it achieves superior module quality and superior coverage of functionalities, unlike the approaches with reduced outputs. We would like to mention that none of these approaches have been designed to support such workflows in the first place (see the supplementary materials File S1 for a detailed description of their methodologies) and that it would be interesting to see whether their module definitions can be used for such exhaustive searches when attuned accordingly. Here, we compare the specific *DECOB* collections with the specific collections that result from filtering the output of the benchmarking competitors for modules which are enriched with a GO term of particular interest in order to demonstrate that reduced, unspecific collections are not appropriate. The reliability of the *DECOB* output for GO term specific modules is not only provided by its excellent ER and COV, but also its superior IC values (see the *DECOB* row in Table 1 and 2). The IC value in particular gives evidence that more functionalities per gene will be covered in general. Hence our specific collections will give rise to comprehensive predictions of very high reliability.

We display a detailed analysis of the collection of modules which resulted from two GO term specific function prediction performances, one in Human and one in Yeast. In Yeast, we focused on GO term “GO:0006333, chromatin assembly” whereas in Human we focused on GO term “GO:0060070, Wnt receptor signaling pathway through beta-catenin”. Our choice of GO terms was motivated by our own research interests. While “Wnt receptor signaling pathway through beta-catenine” plays an important role in development, “chromatin assembly” is critical for regulating gene expression. We collected modules from all methods under consideration, by selecting only those which were enriched with one of the two GO terms. Subsequently, we analyzed these collections.

#### Yeast: GO:0006333, Chromatin Assembly

*DECOB* provided us with 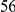 modules which were enriched with genes associated with chromatin assembly. These 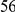 modules contained on average 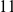 genes and had an average overlap of 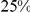.

**Figure 3:**
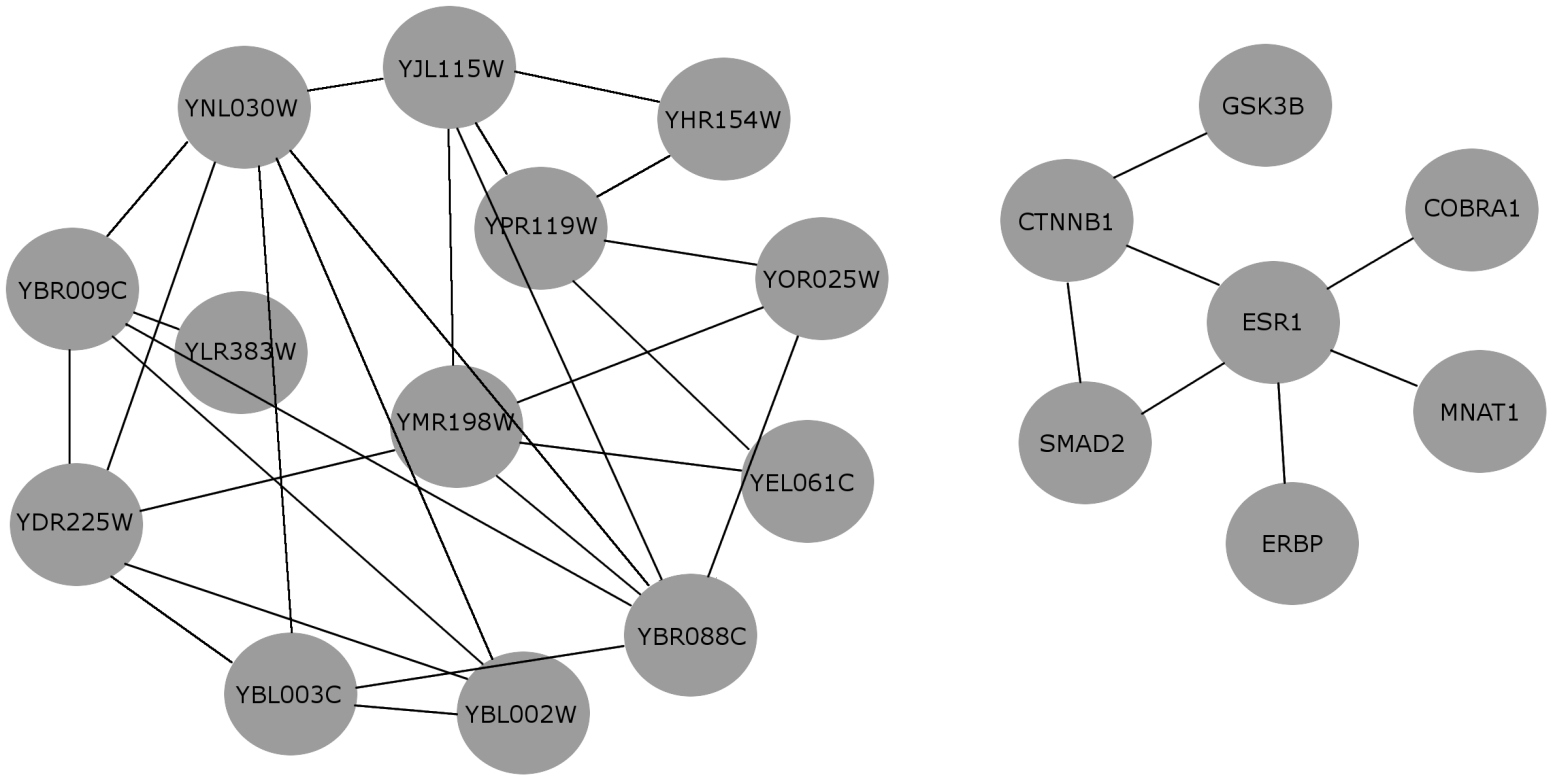
Two real case examples of a Yeast (left) and a Human (right) module as inferred by application of *DECOB* and further filtering by GO terms of specific interest. The Yeast module on the left was obtained by screening the output of *DECOB* for modules which are enriched with the GO term “Chromatin Assembly” (GO:0006333). The Human module on the left was obtained by screening the output of *DECOB* for modules which are enriched with the GO term “Wnt Receptor Signaling Pathway through Beta-Catenin” (GO:0060070).

As a first point, our analysis revealed interesting interrelationships in the *DECOB* modules. Note that we can compute a ranking of the modules, as is described in the Methods section, subsection “DECOBRA: Algorithm”. We found that the module which was top-ranked among the 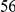 modules carried particularly interesting, potentially novel, information about chromatin assembly, see Figure 3.

In more detail, this module presents 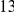 members that function in chromatin structural modification. Five members encode histone subunits: HTB2 (YBL002W), HTA1 (YDR225W), HTA2 (YBL003C), HHF1 (YBR009C), and HHF2 (YNL030W). Recall that histones are core proteins that DNA wraps around to form nucleosomes. Histones, especially the tails, can be modified to form euchromatin or heterochromatin structures which are commonly associated with transcriptionally active region and transcriptionally silent region, respectively. HST3 (YOR025W) is an example of deacetylase that removes acetyl groups from histones (specifically H3K56) to promote formation of heterochromatin [35], [36]. HST3 works in concert with RTT107 (YHR154W) and other proteins to establish transcriptional silencing in locus such as HMR, HML, and telomeres [37]. ASF1 (YJL115W) also facilitates gene silencing by promoting nucleosome assembly by chromatin assembly factor I (CAF-I) [38], [39]. This notion is supported by yeast strains with mutation in ASF1 show defects in heterochromatic gene silencing [40]. ASF1 binds acetylated form of histones and stimulates nucleosome assembly in an HIR and POL30 (YBR088C) dependent manner [39]. The mechanism and interaction between ASF1, HIR proteins, and POL30 is still unclear. Heterochromatin assembly, kinetochore formation, and chromosome segregation is a tightly linked process. SWI6 in S. pombe functions in gene silencing, kinetochore assembly, and microtubule attachment to kinetochores [41], [42]. Similarly, CAF-I and HIR proteins in S. cerevisae, which are important for heterochromatin assembly, also function in kinetochore assembly [43]. Other proteins that have a role in this coordinated process include SMC5 and SMC6 (YLR383W). SMC5-SMC6 complex are localized to centromeres and are crucial for proper chromosome segregation both in S. pombe [44], [45] and in S. cerevisae [46], [47]. It is therefore no surprise that kinesins CIN8 (YEL061C) and CIK1 (YMR198W) are also members of the module where they are crucial for structural integrity of mitotic spindle during mitosis when chromosomes segregate [48]. CIN8 and CIK1 are readily degraded by CDH1. CLB2 (YPR119W) activates a mitotic kinase CDC28 to inhibit CDH1 to allow accumulation of CIN8 and CIK1 [49]. Taken together, all 13 members of this module are reasonably grouped. Our study here suggests histone and histone modification proteins work in a concerted effort with kinetochore proteins and kinesins during mitosis. This has been shown to some extent with CAF-I and HIR proteins [43]. According to our module, ASF1, which functions together with CAF-I and HIR, may also function in kinetochore formation and chromosome segregation during mitosis.

An analysis of the modules of the other methods revealed that *DECOB* is the only method that makes such prediction. In general, the modules generated by *DECOB* are not found by any of the other methods. Moreover, the modules from other programs with GO-term enrichment in chromatin assembly/disassembly show limited overlap with the *DECOB* modules.

Conversely, the other methods predict genes to be associated with chromatin assembly or related processes which cannot be found in any of the *DECOB* modules. The *COC* modules contain 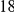 genes where 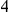 are Histone genes, 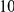 are ribosomal proteins, and the rest are membrane associated or membrane transport protein. The relationship between the members of this module in terms of chromatin assembly/disassembly does not become obvious. Similarly, the *Matisse* modules do not present obvious relationships in terms of chromatin assembly/disassembly. Here, out of 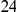 genes in total, 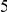 are histone genes, 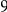 are ribosomal proteins, and some genes involved in RNA processing and amino acid degradation. Apart from *DECOB*, the *MCL* module presents the most plausible predictive quality. It consists of polymerase, topoisomerase, and DNA repair genes. However, *MCL* only generates one module, consisting of 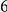 genes. The histone genes are clearly missing in this module. Lastly, one *SAMBA* module (cluster 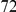) shows high overlap (among all enriched *SAMBA* modules) with the top-ranked *DECOB* module we analyzed. However, while being of high overlap, it also has genes not directly related to chromatin assembly/disassembly such as genes involved in nuclear export, mRNA localization, Golgi membrane protein, and zinc transporter protein.

Last, the *DECOB* modules are generally better enriched in terms of 
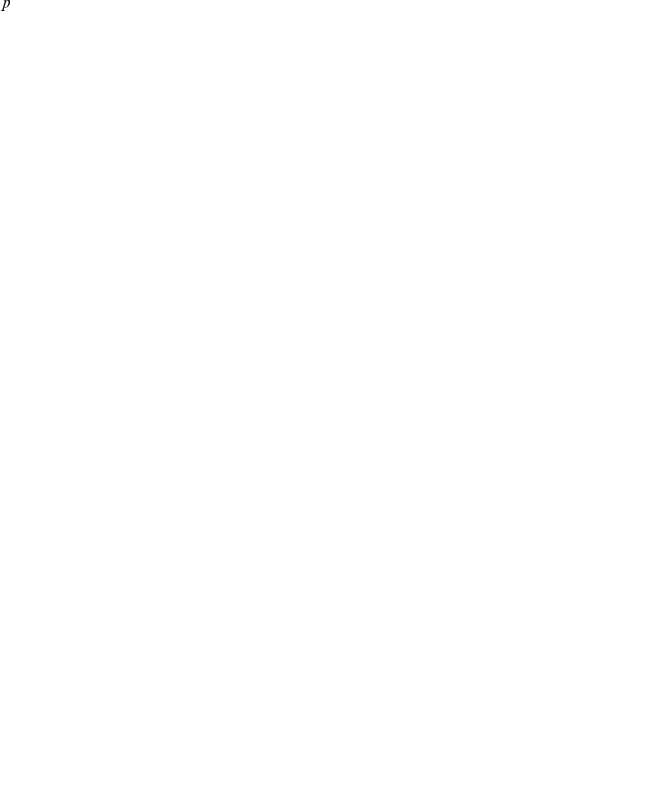
-values.

#### Human: GO:0060070, Wnt Receptor Signaling Pathway through Beta-Catenin

*DECOB* delivered 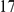 modules which were enriched with genes associated with GO term GO:0060070. These 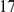 modules contained 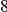 genes on average and had an average overlap of 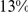. In the following, we focus on analyzing the *DECOB* module which was most significantly enriched since it carried particularly interesting interrelationships. We will comment on its contents in more detail in the following, before turning our attention to the modules of the other methods. See the right of Figure 3 for a picture. Note already now that only *COC* and *MCL* returned modules which were enriched with GO:0060070.

The selected *DECOB* module consists of 7 genes: COBRA1, CTNNB1, ERBP, ESR1, GSK3B, MNAT1, and SMAD2. CTNNB1 and GSK3B are known members in 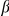-catenin signaling. CTNNB1 (also known as 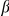-catenin) is a key component in Wnt signaling that is able to translocate to the nucleus to modify many transcription factors such as lymphoid enhancer factor (LEF) [50] and FOXO transcription factors [51]. GSK3B regulates CTNNB1 level by phosphorylating CTNNB1 for degradation [52]. SMAD2 is a member of the TGF-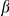 signaling pathway. The remaining 4 genes (COBRA1, ERBP, ESR1, and MNAT1) are part of the estrogen receptor pathway. ESR1 (estrogen receptor 1) is a ligand-activated transcription factor that binds to the estrogen-response element (ERE) while ERBP (estrogen receptor binding protein) binds to and enhances ESR1 activity. ESR1 activity is regulated by a number of factors. COBRA1 interacts with ESR1 and is also able to inhibit ESR1 target gene activation upon estrogen stimulation [53]. Similarly, MNAT1 also interacts and translocates with ESR1 upon estrogen activation [54]. It is suggested that ESR1 activity may be influenced by MNAT1 via chromatin remodeling [54]. It is only recently that we begin to see some evidence suggesting the convergence of the estrogen receptor pathway and Wnt signaling pathway. Kouzmenko et al. showed in Drosophila that ER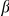 (ESR1) functionally interacts with 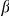-catenin and that 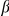-catenin can be recruited to EREs [55]. Mendez et al. similarly showed that GSK3 positively regulate estrogen receptor activity in N2a cells by enhancing transcription of target genes [56]. Having this in view, the *DECOB* module under consideration presents some interesting predictions of potential novel interactions between Wnt signaling pathway and estrogen receptor pathway.

Another interesting feature was to observe that overlap, ESR1 was found to participate in *DECOB* modules, different from the one under consideration here, which were enriched with the GO term “Estrogen receptor pathway”, but not with the GO term under consideration here (“Wnt signalling pathway through 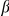-catenine”). This is a concrete example of the benefits of overlapping modules, which, in this example, share ESR1 as a member, but reflect different functionalities.

None of the modules of the other methods make such predictions. Aside from *DECOB*, *COC* and *MCL* are the only two programs that return a module which is enriched with GO term GO:0060070. None of the above methods generates a module from the *DECOB* output. The only *COC* module contains 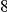 genes. While some of them are for DNA repair (MRE11A, POLI, RUVBL2), one is associated with microtubule regulation (MAPRE1). It is not obvious at the point if any cross talk occurs between Wnt signaling pathway and DNA repair. *MCL* on the other hand yields a 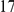 gene module that is highly enriched in cell adhesion and junction proteins such as cadherins (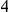 genes), catenin (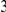 genes), desmosomes (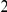 genes), and their associated proteins (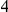 genes). Some of these genes, like 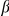-catenin, have a membrane associated form functioning in cell-cell contact and a cytoplasmic form to function in signaling pathway but the idea that all adhesion molecules also play a role in Wnt signaling is currently not supported by the literature. At any rate, there are similar *DECOB* modules, predicting similar contexts as the *MCL* module. Note, however, that the *DECOB* modules show higher significance in GO terms relating to cell-cell junction than Wnt signaling.

In summary, our analysis reveals that the GO-term specific collection of *DECOB* modules possesses the better predictive power, since it reveals well-known, relevant and predicts plausible, interesting relationships that other methods miss.

## Discussion

In the Introduction, we outlined that despite the great advances in the area of functional module discovery which were made in the post-genomic era, a few issues whose overcoming promised further potentially significant improvements had remained unresolved.

First, the tractability of the computational problem to exhaustively search for *densely connected biclusters*, that is, dense and connected regions in interaction networks where genes are sufficiently co-expressed had remained an unresolved issue. However, the idea that densely connected biclusters reliably reflect functional modules was widely supported and well-established (e.g. [5], [6], [8], [9]). Beyond the cited evidence, it is interesting to notice that *MCL* which operates on network data only and performed quite favorably in a comparative study [23], employs a definition which is akin to that of densely connected regions in the interaction networks (see the supplementary materials File S1 for a detailed description of *MCL*). However, none of the approaches which operate on both interaction network and gene expression data, explicitly addresses this objective. Also, while there is evidence in the literature that dense connectivity gives rise to reliable modules in genetic interaction networks [5], [7], none of these approaches were evaluated on such data.

Second, combined approaches tend to partition the datasets, thereby establishing one-to-one correspondences between genes and functionalities although it is well-known that genes can participate in several functional contexts. Note that methods which operate only on gene expression data can infer overlapping modules [57]–[59] which underscores the benefits of this idea.

Third, there were no approaches which generated large, comprehensive collections of high-quality modules resulting from exhaustive screens of the modular organization of organisms. The idea behind such exhaustive searches is to subsequently tailor the resulting large collections to more specific needs, by means of fast filtering strategies. Apart from convenience in such annotation-specific module discovery tasks, exhaustive collections may also provide a global picture of the modular organization of an organism.

In this article, we presented an algorithmic framework with which to resolve the outlined issues. The framework is centered around the problem of exhaustively searching for *densely connected biclusters* using the property of antimonotonicity. The framework outputs a collections of densely connected overlapping biclusters. No densely connected bicluster is missed by our procedure which results in a large, comprehensive collection of high-quality modules.

In order to demonstrate the benefits of our approach, we tested two module discovery methods, *DECOB* and *DECOBRA*, which arise from our framework. The output of *DECOB* results from merging densely connected biclusters which share a significantly large overlapping core. The output of *DECOBRA* results from further reducing the output of *DECOB* according to a ranking-based filtering procedure. This procedure serves the purposes of a fair competition—the output of *DECOBRA* is comparable to the outputs of existing approaches in terms of numbers of modules and of genes covered. We then employed *DECOBRA* in a standard benchmarking procedure. The comprehensive output of *DECOB* was employed to predict functional relationships of particular interest using two examples. For this purpose, the output of *DECOB* was filtered according to the particular interests as specified by two GO terms.

In the benchmarking competition, *DECOBRA* proved to be superior over the state-of-the-art approaches under consideration. While this is good evidence of that densely connected biclusters indeed reliably reflect functional modules, we observed some further interesting phenomena:

1. Our baseline method, which operates on interaction network data only, achieved respectable enrichment (ER) values (see Table 1 and 2), which underscores that connectivity is a valuable concept when screening interaction network data. This also shows that achieving enrichment up to 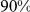 does not require elevated levels of sophistication. However, the fact that it also achieves respectable coverage values is quite disturbing and casts certain doubts on enrichment and coverage as the only measures to assess the performance of module discovery programs. As a first attempt to mend these deficiencies we introduced IC which reflects how many functionalities per gene are covered on average. It is interesting to observe that the baseline method achieves only poor values here, whereas the sophisticated module finder *MCL*, which also operates on interaction network data only, achieves superior values in these novel categories while being at most on a par with the baseline method in the standard values.
2. We observed that *MCL* achieved good coverage values (COV and IC) while achieving only relatively low (below 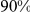) enrichment (ER). This reflects that, on one hand, quite a substantial percentage of functional contexts is reflected by interaction data. However, on the other hand, still a significant amount of dense and connected regions do not reflect modules, likely due to the fact that the underlying combinations of edges are not simultaneously present within cellular contexts. In other words, interaction network data is *static*.
3. *SAMBA*, which operates only on gene expression data, achieves good (even superior enrichment values in the human data set) without achieving good overall coverage. This reflects that coherent expression patterns indicate modular arrangements when co-expression is appropriately modeled. However, not all modular arrangements become visible at the transcriptional level which is a well-known fact [4].
4. The essence of the previous points is that a good idea for module discovery approaches is to employ the comprehensive predictive power of interaction network data while using gene expression as a control element. Thereby, one must be aware of that state-of-the-art definitions of co-expression (such as the one of *SAMBA*) could rule out too many network patterns, which would again result in low coverage values. Note that the definition of co-expression of our approach is a rather relaxed one. To our understanding, the combination of network and expression criteria as per our approach explains both the superior enrichment and superior coverage.

Note finally that, independent of the fairness issues (*DECOB*'s output is larger by one order of magnitude than the outputs of the benchmarking competitors), *DECOB* achieves the best values in all benchmark measures. We filtered the output of *DECOB* by specifying GO terms of interest and studied the resulting real case examples of module collections. By doing so, we aimed at demonstrating that the large output of *DECOB* can be employed to more conveniently tackle more specific function prediction tasks. A thorough analysis of the specific collection of *DECOB* modules and the (much smaller) collections of the prior approaches revealed that the *DECOB* modules possess the greatest predictive power, since it reveals well-known, relevant and predicts plausible, interesting relationships that other methods miss. It would be interesting to see how related approaches perform when being tailored to address such tasks. However, it remains unclear to what extent the comparison partners considered here (see the supplementary materials File S1 for detailed descriptions of the approaches) can be modified to support such tasks.

In summary, we have provided evidence of the substantial benefits of our module discovery framework when it comes to resolving the issues outlined in the Introduction. Future work will be concerned with adapting our methodology to confidence-scored interaction data, which has received considerable attention in the recent past. Moreover, we are planning to explore the applicability of gene co-expression constraints which are different from that of Definition 1. For example, there usually is a negative correlation between genetic interacting genes belonging to alternative pathways. As such, order preserving submatrix analysis is a promising direction as it can handle both positive and negative correlation [16]. Last, mining modules with density thresholds that are related to module size in the style of [60] should be beneficial. Note that in [60], the determination of significance thresholds for subgraph size dependent density is incorporated into a mining algorithm which, in contrast to our approach, partitions the networks into a fragmentary collection of subgraphs hence outputs an incomplete, non-overlapping collection of modules. Combining a subgraph size significance analysis with an exhaustive search for densely connected biclusters should yield further improvements in module discovery.

## Methods

### Data

#### Yeast

We extracted the interaction network, containing both PPI and GI interactions from multiple publicly available datasets from the BioGRID database [61]. Gene expression data was given by the yeast compendium dataset [62]. It reports fold changes of experiment against control in as many as 300 cDNA experiments. We discarded genes whose ratios were to be found in a 1.5 times variance interval around the mean over all conditions, hence nowhere exhibited significant expression levels. This amounted to 1043 differentially expressed genes with 2664 interactions in the resulting network.

#### Human

Again, the PPI/GI network was downloaded from the BioGRID database [61]. For the gene expression data, we used the comprehensive human tissue expression dataset [63], which lists fold changes over 115 cDNA experiments across 35 different tissue types. In order to account for activity, we only retained variably expressed genes which were with at least 2-fold ratio variation from the mean in at least two samples, as suggested by the authors of [63]. As a result, the human dataset contained 3628 genes connected by 8924 interactions in the respective network.

### General Strategy

On a high level, our method consists of the following steps:

1. Infer the entirety of all densely interconnected subgraphs whose genes are co-expressed (definitions see subsection “Densely Connected Biclustering: Problem Definition and Properties” below), *DECOB* algorithm, see subsection “DECOBRA: Algorithm” below.
2.
  - In order to provide specific collections of modules, specify the functionality of interest and filter the (comprehensive) output accordingly (results see Results section, subsection “Advantage of Exhaustive Searches”)
  - In order to obtain a small and reliable collection of modules which is independent of choices of GO terms, we apply a ranking procedure that ranks the modules according to density and coherence in expression. We then select modules using these rankings as a guide, *without* that numbers of modules have to be specified beforehand (*DECOBRA*, see subsection “DECOBRA Algorithm” below).

### Densely Connected Biclustering: Problem Definition and Properties

In order to formally introduce our problem definition, we will employ the following terminology.

A **profile network** is defined as an undirected graph 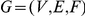 consisting of a node (gene) set 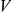, an edge set 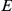 and a **profile function**

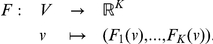


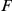
 assigns a fold change expression profile to each node of an interaction network. For 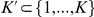, we will refer to 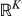, the projection of 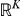 onto the dimensions specified by 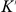, as a **profile subspace**. We are interested in the following three properties of an induced subnetwork 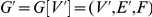: co-expression, density and connectedness, which are summarized in the following definition.

### Definition 1 (Densely connected Biclustering)

*Let* 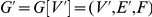 *be an induced subnetwork of a profile network* 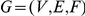.

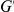
 *is* ***co-expressed*** *wrt. (with respect to)* 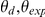 *if there is a profile subspace* 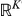, 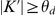 *such that for all* 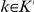


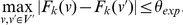

*This translates to that the expression levels of genes* 
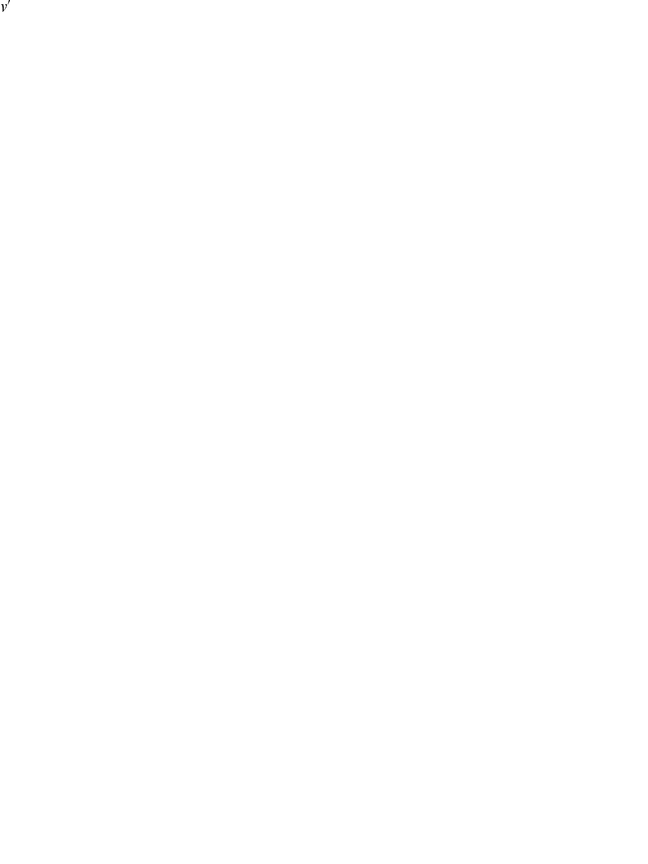
 *of the subgraph* 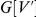 *do not differ by more than* 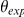 *under the at least* 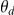 *many cellular conditions indexed by* 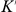. *Note that a set of correspondingly co-expressed genes can be viewed as a* **bicluster** *of genes and conditions in the sense of the usual definition of a bicluster.*
2. *The* ***density*** *of* 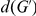 *of* 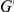 *is defined as the ratio of the number of edges in* 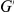 *over the number of possible edges in* 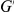,
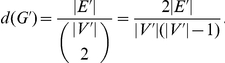

*We say that* 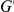 *is* 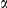 **-dense** *if*

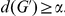

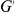
 *is* ***connected*** *if there exists a path in* 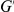 *between any pair of nodes in* 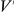.

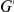
 *is called a* ***densely connected bicluster (DCB)*** *or, equivalently, satisfies the* 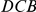 **constraints** *wrt*. 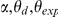 *if* 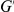 *is connected, co-expressed wrt*. 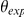 *and* 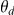 *and* 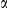-*dense*. *A* 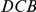 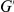 *is* **maximal** *if it is not a proper subgraph of another densely connected bicluster.*

See Figure 1 for an example of a densely connected bicluster.

#### Biological Instances

In the instances of profile networks 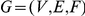 considered, 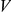 is a set of genes/gene products and edges 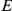 correspond to both PPI and GI interactions. 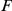 can be identified with fold change expression profiles of the genes. Accordingly, a 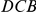 will be a set of genes that are co-expressed within a 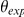 fold-change neighborhood of each other across at least 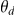 experimental conditions and whose associated nodes form a densely connected interaction subnetwork (see Figure 1).

### Definition 2 (Densely Connected Biclustering (*DCB*) Problem)

#### Input

*Profile network* 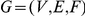, *density threshold* 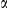, *homogeneity threshold* 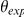 *and minimum number of dimensions* 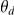.

#### Output

*All maximal* 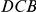 *s of* 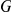 *satisfying the* 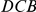 *constraint specified by* 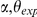 *and* 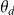.

The 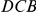 problem is 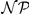-hard. Its decision version is 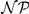-complete, shown by a simple reduction from the max-clique problem [64]. As a straightforward observation note that naive approaches to the 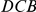 problem would require an exhaustive enumeration of all 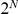 subnetworks of 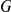, which is infeasible in general (here, 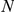 will be on the order of the number of genes in an organisms hence on the order of several thousands). In case of PPI/GI networks, tractability is provided based on the following observation.

### Definition 3 (Loose Anti-Monotonicity)

*A constraint is called* ***loose anti-monotone*** *if for each network* 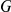 *of size* 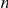 *that satisfies the constraint, one can find at least one induced subnetwork* 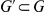 *of size* 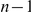 *satisfying the constraint.*

The crucial observation for rendering the search problem tractable is that the 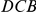 constraints are loose anti-monotone if 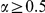. Below we provide a proof sketch for this to hold. Detailed definitions and fully elaborated proofs can be found in [33] and [11].

#### Proof Sketch

Clearly, the co-expression constraint holds for all induced subnetworks of size 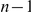 of a 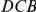 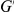 of size 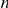. We can therefore restrict our attention to dense connectivity. To obtain a 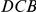 of size 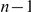 of 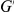, one tries to remove the node (and with it its edges) whose degree is smallest. We will be done if the resulting network is still connected. If the network is disrupted into two sets of nodes, then the smaller one of the components, including the disrupting node, contains at most half of the nodes of the original network. This translates to that the degree of these nodes, divided by the number of possible incident edges (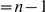) is at most 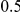. Therefore, some straightforward computations reveal that one can remove all these nodes without violating the density constraint. It remains to observe that removing a node in the smaller component that is farthest away (in terms of shortest paths) from the disrupting node will not disrupt the network.

We would finally like to point out that for 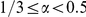 the 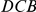 constraints are not loose anti-monotone. By means of further theorems and, based on them, additional subroutines that follow the core routine from below, we would have been able to infer all 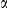-dense 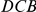
s also for 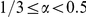 (see [11], [33] for details).

In order to have an appropriate choice of 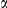 we examined the densities of the Yeast protein complexes and pathways. See subsection “Choice of Parameters” below for a more detailed description.

#### Related Work

A most recent approach whose theoretical framework supports inference of all dense, but not necessarily connected subgraphs in interaction networks (without particularly addressing gene expression) is [9]. While they employ their methodology to only search for connected, dense subnetworks, the algorithmic strategy does not guarantee to do this exhaustively and one can show that they miss certain dense and connected subnetworks. The idea of mining for densely interconnected subgraphs was also successfully applied to co-expression networks where edges connect genes when they are significantly co-expressed across a range of different cellular conditions. In this case, several specifically adapted heuristics were devised to tackle the corresponding search problems [65], [66].

### DECOB Algorithm

**Figure 4:**
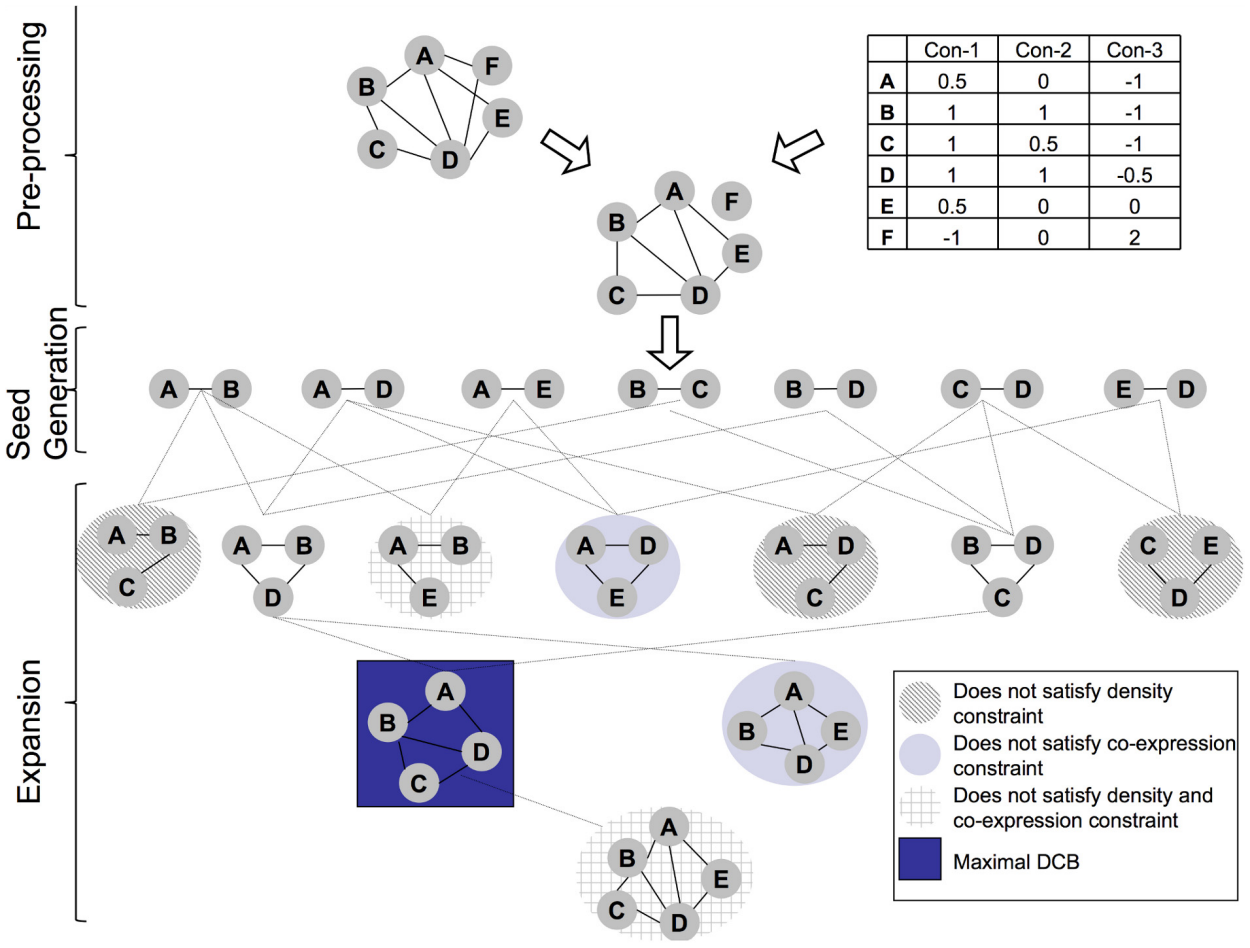
Illustration of the *DECOB* algorithm on a simplified example consisting of six genes and three gene expression conditions. *DECOB* constraints are specified by: 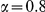 (density), 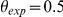 (maximum difference in expression) and 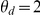 (number of expression conditions). The algorithmic strategy is to traverse the lattice of all subnetworks in a breadth-first fashion. Any subnetwork which is not a densely connected biclusters can be discarded due to that every densely connected bicluster necessarily has a densely connected bicluster as a parent ( = subnetwork contained in the original one, see definitions 1 and 3 and the surrounding discussions). For esthetical reasons, we have omitted B-C-D-E although, as a child of the densely connected bicluster B-C-D, it is also examined. B-C-D-E, just as A-B-D-E will be discarded since it violates the density constraint.

The core strategy of *DECOB* is to narrow down the huge search space consisting of the 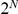 (where 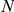 is the number of the nodes of the network, i.e. the number of genes in an organism) subnetworks of the original network by means of the loose anti-monotonicity of the *DCB* constraint. See Figure 4 for an example. In a preprocessing step, we remove edges whose nodes refer to genes that are not sufficiently co-expressed, that is edges between genes whose expression profiles do not meet the co-expression constraint, as such edges cannot participate in any *DCB* (note that the co-expression constraint, taken by itself, is strongly anti-monotone which means that none of the children of such gene pairs can meet the co-expression constraint). Then we conceptually organize all connected subnetworks in a hierarchical structure (formally a lattice) where a subnetwork is a child of another one if it can be obtained by adding exactly one gene (and the corresponding edges) that is connected to the parent subnetwork. Note that a child is larger than its parent which may be a bit counterintuitive.

We then traverse this structure top-down, in a breadth-first search. This means that subnetworks of size 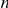 are only checked upon having produced all *DCB*s of size 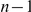. The point is that when it comes to examining subnetworks of size 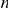, we can restrict ourselves to checking children of *DCB*s of size 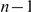, as the loose anti-monotonicity of the *DCB* constraints guarantees that every *DCB* of size 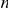 necessarily has a *DCB* of size 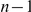 as a parent. For example, in Figure 4 only children of the *DCB*s A-B-D and B-C-D are examined further whereas other subnetworks (e.g. A-B-C-E) are not checked as they have no *DCB* as a parent. If a 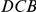 cannot be expanded by a node (i.e. it is *maximal*) it is returned as an output. The only maximal *DCB* in Figure 4 is A-B-C-D.

In order to both increase and adequately evaluate the biological quality of our modules we subsequently employ a refinement procedure. Its biological motivation is that, in biomolecular networks, functional subunits often consist of a *dense* core in combination with genes which are “attached” to the core (e.g. [32]). The following refinement procedure will merge 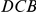
s when they overlap to a high degree. Ensembles of genes resulting from this merging procedure reflect such “core-attachment” constellations. In addition to such motivation, missing data is another issue that we address by the refinement procedure.

We iteratively merge pairs of 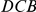
s if they overlap in at least 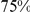 of their members as well as in at least 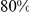 of their associated co-expression subspaces (referring to the gene expression conditions under which they are sufficiently co-expressed). This is because currently available PPI/GI networks are far from being complete as well as that gene expression experiments contain a high amount of noise. These issues result in significant amounts of modules that are split up into fractions. The refinement step alleviates this problem by relaxing the density and co-expression constraints in such cases. Note that the refinement procedure implies that, despite our choice of a density threshold of 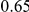 (see below), the density of the inferred modules can be lower than 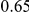.

Throughout the article, we refer to the modules which result from merging 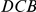
s as described above as *DECOB* modules.

### Choice of Parameters

In order to choose 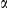 appropriately we examined the average density of the Yeast protein complexes and the pathways as downloaded from the SGD database [31]. See Figure 1 in File S1 for corresponding statistics. While the mean density of those complexes was found to be 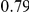, we found that the average density of the annotated modules was reduced upon combination of the two datasets and subsequent removal of nodes which referred to genes with missing gene expression data. Therefore, we chose 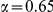 as a biologically well motivated density threshold and, based on the underlying biological inspiration and the good results we obtain, we suggest this choice of parameter as a default value. Note that the density of our modules can become lower than 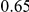 upon treatment in the postprocessing step.

Similarly, based on the distributions of the number of co-expressed dimensions of the annotated modules, we further chose 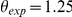 and 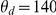 (out of 300) for the yeast dataset. Contrary to yeast, there is no comprehensive true human module dataset. With regard to the fact that the human expression dataset contains a high amount of missing values (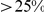) which adverts to a high amount of noise, we used more relaxed thresholds (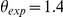, 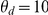 (out of 115)).

### DECOBRA Algorithm

In order to provide a competitor which meets the purposes of the standard benchmarking procedure, we developed a filtering procedure which is based on a ranking of the output of *DECOB*. Thus, the output of *DECOBRA (DEnsely COnnected Biclustering RAnked)* results from subsequent filtering of the output of *DECOB*, as described below.

#### Ranking: Co-expression Ranking

To assess the significance of the co-expression encountered in the output modules, we randomly sampled 2000 connected networks from the instances at hand (see the Results section). We fitted the resulting statistics on numbers of co-expressed conditions to a truncated normal distribution which provided us with a 
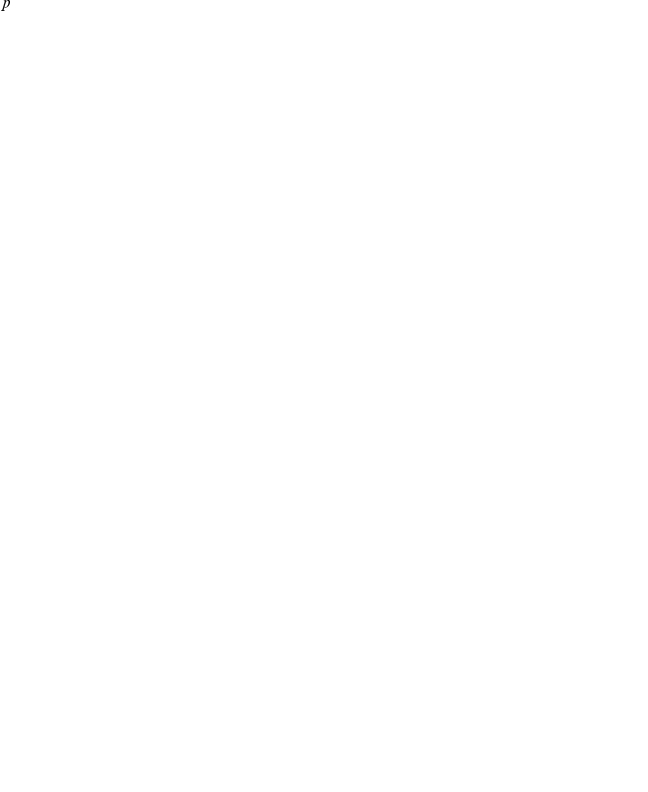
-value for the *DECOB* under consideration.

#### Ranking: Dense Connectivity Ranking

Let 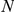 be the number of nodes and 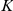 be the number of edges in the complete network under consideration. We interpret the probability that a subnetwork of size 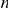, sampled randomly from the network, has 
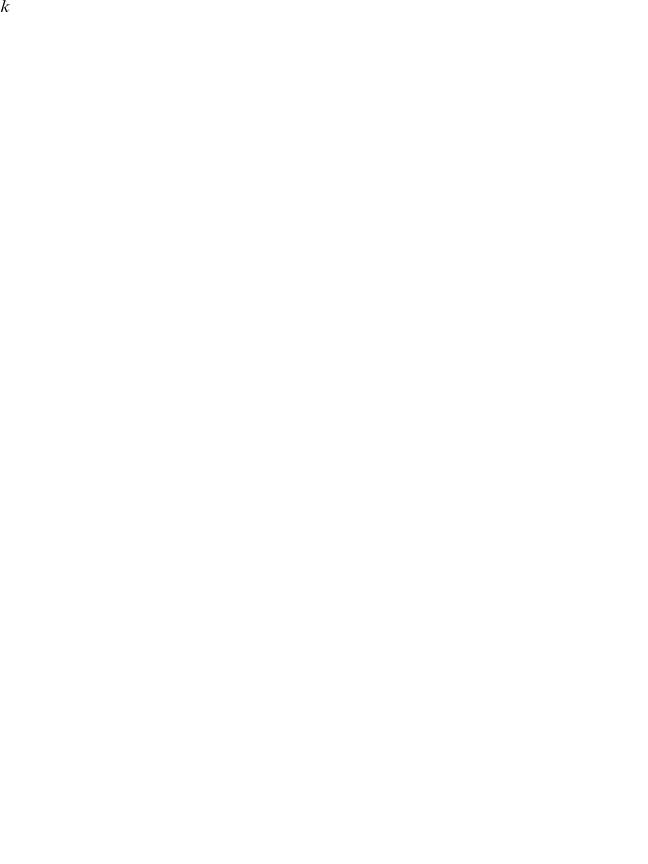
 edges as the corresponding probability of the hypergeometric distribution (which, as a toy description, refers to sampling 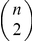 red balls from an urn with 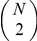 balls 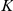 of which are red). We are aware of that counting subgraphs in biomolecular networks and/or respective statistics are areas of active research (e.g. [67]–[69]). The hypergeometric distribution is in accordance with the analyses displayed in [60] hence represents a reasonable choice

#### Overall Ranking and Filtering

We ranked the modules according to both co-expression and dense connectivity separately and used the average of the rankings as an overall ranking. This yields a ranked list of the output of *DECOB*. In order to filter *DECOB's* output accordingly, we traversed the ranking list from top to the bottom and removed all modules whose genes were contained in the modules higher up in the ranking list. The remaining modules are the output of *DECOBRA*. In order to obtain even smaller outputs we suggest to select only the 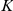 best ranked modules from the output of *DECOBRA* since this yields both high-quality and non-redundant collections of modules. See also Table 1 and 2 for module statistics on such smaller collections (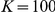 and 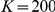).

## Supporting Information

File S1 Detailed description of the algorithms / algorithmic methodologies of the benchmarking competitors of the main paper for better evaluation of the results in the main paper.
(0.08 MB PDF)

Changes compared to our original PLOS One submission (0.44 MB PDF)
