# Supplementary material for: Module Discovery by Exhaustive Search for Densely Connected, Co-Expressed Regions in Biomolecular Interaction Networks
Source: PLoS One. 2010 Oct 25;5(10):e13348. doi: 10.1371/journal.pone.0013348 (PMC2963598; doi:10.1371/journal.pone.0013348)
Supplement: File S1 — Detailed description of the algorithms / algorithmic methodologies of the benchmarking competitors of the main paper for better evaluation of the results in the main paper. (0.08 MB PDF) [file pone.0013348.s001.pdf]

Module discovery by exhaustive search for densely connected, co-expressed regions in biomolecular interaction networks: Supplementary Materials

Recep Colak, Flavia Moser, Jeffrey Shih-Chieh Chu,
Alexander Schönhuth, Nansheng Chen and Martin Ester

September 12, 2010

Abstract

Flexible module discovery by exhaustive search for densely connected, co-expressed interaction subnetworks: Figure motivating our choice of density threshold and description of the algorithms of the benchmarking competitors.

Algorithms of the Benchmarking Comparison Partners

Here, we describe the algorithmic ideas of the comparison partners of subsection 3.1 of the Results section of the main paper in more detail. This is to provide the interested reader with a deeper analysis of the pros and cons of the methods under consideration.

As outlined above, the comparison partners include two integrated methods, one of which is a seminal approach and the other one is the current state-of-the-art approach. Moreover, we selected two methods that operate on a single data type (either interaction network or gene expression data), both of which can be considered to have established the gold standard on the types of data under consideration here. In the following, we particularly address to what extent they address the issues of module overlap and network density which are central issues of our study. In our experiments, we used the recommended parameter settings for all algorithms.

SAMBA (Statistical-Algorithmic Method for Bi-Clustering) (7) is a widely used biclustering algorithm. It can be employed very flexibly and allows to integrate also other types of data. We employed it to gene expression data alone as this has been its traditional domain of applications so far another reason being that it performed comparable, if not better, in a recent comparative study of such biclustering algorithms (6). In general, biclustering algorithms are the prevailing methodology when it comes to analyzing gene expression data resulting from multiple cellular conditions.

Algorithm: Given a set of genes V and a set of cellular conditions U, SAMBA constructs a bipartite graph $G = (V \cup U, E)$ where there is an edge $(v, u) \in E$ between gene v and condition u whenever gene v exhibits a significant change in expression under condition u. Based on this data arrangement, (7) present an algorithmic toolkit with which to infer combinations of genes and conditions which are significant in terms of the edge patterns found within this subgraph compared to the overall arrangement of edges. Such "heavy" subgraphs are subsequently filtered in order to reduce the overlap between them. Basically, it finds sets of genes that jointly respond across a subset of experimental conditions in a gene expression data set.

Overlap: Resulting modules do overlap. To our understanding of the method, the amount of overlap can be flexibly controlled.

Network Density: Not addressed, since SAMBA does not take into account network data.

Output Characteristics: In accordance with the general observations on modules inferred from gene expression data alone, SAMBA yields collections of modules that are highly reliable in terms of GO term enrichment. In general, the modules are quite large such that many genes are covered. However, the modules do not reflect many functional contexts. Resulting modules do not refer to dense subnetworks in the available PPI/GI data.

MCL (Markov Clustering) (3; 5) only considers network data. It outperformed other methods of this type in a recent comparative study (1). It was originally used to successfully screen protein databases for protein families (3).

Algorithm: The concept that underlies MCL is to model the network data as a matrix where both rows and columns are the genes under consideration. The entries of the matrix then are the weight of the edge that connects the two genes in the network if there is one and zero otherwise. If edges have no weights the entry is set to be one if there is an edge and zero otherwise. We had to deal with the latter, unweighted case.

Subsequently, MCL computes powers of the matrix and its entries alternately until no further changes can be observed. Rearranging the rows and the columns of the resulting matrix results in a block structure where blocks are finally output as modules. As can be proven and is well-known, the iterative procedure of taking powers of both matrix and entries results in blocks which are dense subnetworks, in terms of that random walks starting from one node in the network will, highly likely, soon hit another node in the same block.

Overlap: In theory, the resulting blocks (modules) can be overlapping. However, we did not observe this in our analyses. To our understanding, it is highly unlikely in theory to obtain overlapping blocks in matrices having been obtained from PPI networks, due to their sparsity.

Density: Resulting modules reflect dense subgraphs, since density is clearly correlated to subnetworks that are tight in terms of random walks in the inferred subnetwork. In more detail, tightness means that, given two arbitrary nodes in the subnetwork and starting the random walk from one of them, the random walk will, highly likely, soon hit the other one where the random walk is allowed to proceed through the complete PPI network. This random walk property can be mathematically proven to hold true for MCL subnetworks.

Output Characteristics: Resulting modules are the smallest among all tested methods. The amount of covered functionalities is high while the percentage of enriched modules is rather low. As outlined above, MCL modules are relatively dense, also in terms of the definition we have employed.

Co-Clustering (COC) (4) is a seminal approach on the topic of combined evaluation of large-scale data of multiple types. While having been outperformed by follow-up approaches in the meantime, it is still quite popular, thanks to its clear, straightforward and intuitive design.

Algorithm: The basis of the algorithm is to combine two distance functions between the genes, one reflecting relationships available from the network data and the other one reflecting similarity in expression, into a combined distance function. Subsequently, genes are clustered by means of standard, off-the-shelf clustering algorithms. In more detail, the network distance between two genes is the length of the shortest path between them. The expression distance between two genes g_i, g_j is $1 - Pearson(g_i, g_j)$ where $Pearson(g_i, g_j)$ is the Pearson correlation coefficient, which was suggested in one of the seminal papers

on gene expression analysis (2). The combined distance function is then obtained by first wrapping Subsequently, the single distance functions are wrapped into a logistic function, which reflects a smooth thresholding procedure. The combined distance function is then obtained by simply averaging over the two resulting distance functions. In the original approach, the combined distance function is then used to perform hierarchical average linkage clustering, which is a standard clustering approach, yielding the well known tree-like dendrogram of the genes (see also (2)). Disjoint subsets of leaves (genes) are selected as modules from this dendrogram such that they optimize well known clustering quality measures.

Overlap: Clusters are disjoint, since they correspond to dijoint subsets of leaves in the tree dendrogram.

Density: Density is only marginally addressed by this approach. The idea that two genes have to be connected by sufficiently short paths does not even necessarily yield connected subnetworks as modules. Herefore note that shortest path do not have to lie within the inferred subnetworks.

Output Characteristics: Density is low, clearly resulting from the problem definition. Modules are medium sized. All genes are covered by the modules, since the algorithm partitions the dataset, thereby assigning each gene to a module. This also results in relatively high GO term coverage. However, enrichment is about 70% which is poor, since it does not even attain the enrichment values of randomly sampled connected subnetworks. This also points out that connectivity is an essential necessity to attain good enrichment values.

Matisse (8) is the most recent integrated approach available. It is a probabilistic method that finds connected subnetworks in interaction networks that exhibit high expression similarity. It outperformed all prior approaches in a plausible and fair benchmarking competition which we adopted in this paper as well.

Algorithm: The algorithm relies on information which is incorporated into both an unweighted constraint and a weighted similarity graph. While, in case of the biological instances of interest, the constraint graph simply is the PPI network, the similarity graph reflects relationships obtained from the gene expression data under consideration. The weights in the similarity graph result from a hypothesis test which reflects the likelihood that the connected genes are co-expressed. Genes are then ranked according to their total edge weight in the similarity graph. The algorithm is initialized by greedily picking genes according to their ranking and adding a restricted amount of neighbors such that the neighborhood forms a heavy, in terms of the weights, subnetwork in the similarity graph. In order to perform this selection procedure, it can be necessary to use heuristics. The resulting subnetworks are then improved by testing whether adding, removing or joining two subnetworks improves the overall weight of the subnetwork. Thereby, subnetworks are kept to be disjoint. The final output is computed by applying a filtering procedure, reflecting the significance of the obtained score, to the collection of modules obtained by the procedure from above.

Overlap: Matisse does not return overlapping modules as it is explicitly designed not to do so.

Density: Density is not addressed, neither explicitly nor implicitly, since the focus of this algorithm is to maximize scores based on gene expression data only while simple connectivity is assured as a constraint. In practice, however, the resulting modules are quite dense.

Output Characteristics: Resulting numbers of modules are smallest among all competitors while the size of the modules is relatively high. In terms of enrichment, Matisse can be considered to be superior over all prior approaches. However, its coverage, in terms of enriched GO terms is low; only little functionalities are covered by its output.

To summarize, none of the comparison partners addresses the problem of finding co-expressed, dense subnetworks according to Definition 5.1 in the Methods section. The only method that yields overlapping modules is *SAMBA* whereas the only one that addresses the question of finding dense subnetworks is *MCL*.

Theoretically, *MCL* can also output overlapping modules, but we have not observed this in our analysis. However, *SAMBA* and *MCL* are both not integrated hence more prone to noise or missing data that occur in one data type only. The integrated methods, *COC* and *Matisse*, both do not address finding dense subnetworks and they do not produce overlapping modules. Methods that assign each gene to a cluster (*MCL, COC)*) may also suffer from forcibly collecting proteins into modules which, due to the current amounts of noise in the data, cannot be done reliably. Our method does not suffer from any of the mentioned drawbacks.

References

[1] Brohee S, van Helden J (2006) Evaluation of clustering algorithms for protein-protein interaction networks. BMC Bioinformatics 7:488.

[2] Eisen MB, Spellman PT, Brown PO, Botstein D (1998) Cluster analysis and display of genome-wide expression patterns. Proc. Natl. Acad. Sci. USA 95:14863-14868.

[3] Enright AJ, Van Dongen S, Ouzounis CA (2002) An efficient algorithm for large-scale detection of protein families. Nucleic Acids Research 30:1575-1584.

[4] Hanisch D, Zien A, Zimmer R, Lengauer T (2002) Co-clustering of biological networks and gene expression data. *Bioinformatics* 18 (Suppl. 1):145-154.

[5] Krogan NJ, Cagney G, Yu H, Zhong G, Guo X, et al. (2006) Global landscape of protein complexes in the yeast Saccharomyces cerevisiae. Nature 440: 637-643.

[6] Prelić A, Bleuler S, Zimmermann P, Wille A, Brühlmann P, et al. (2006) A systematic comparison and evaluation of biclustering methods for gene expression data. Bioinformatics 22(9): 1222-1129.

[7] Tanay A, Sharan R, Shamir R (2002) Discovering statistically significant biclusters in gene expression data. Bioinformatics 18 (Suppl. 1): 136-144.

[8] Ulitsky I, Shamir R (2007) Identification of functional modules using network topology and high-throughput data. BMC Systems Biology 1:8.

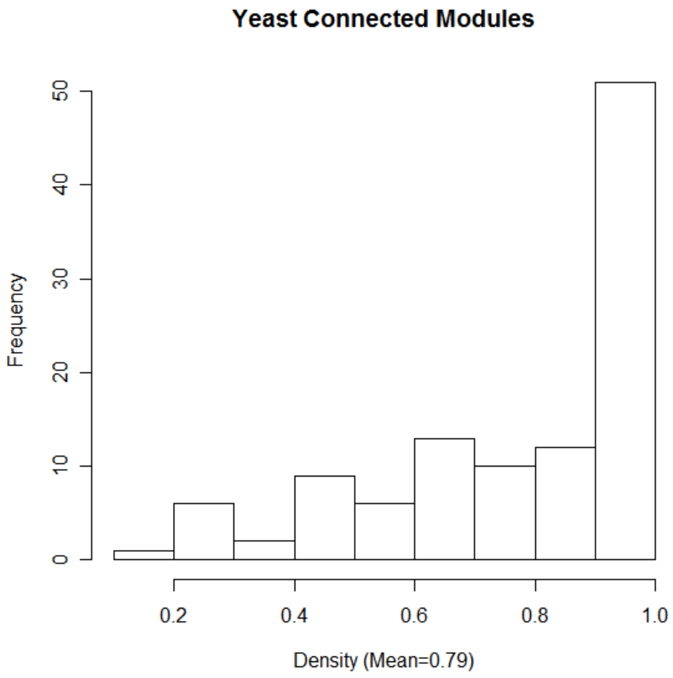

Figure 1: Density distribution of complexes in Yeast.
