# Supplementary material for: Module Discovery by Exhaustive Search for Densely Connected, Co-Expressed Regions in Biomolecular Interaction Networks
Source: PLoS One. 2010 Oct 25;5(10):e13348. doi: 10.1371/journal.pone.0013348 (PMC2963598; doi:10.1371/journal.pone.0013348)
Supplement: Changes compared to our original PLOS One submission — (0.44 MB PDF) [file pone.0013348.s002.pdf]

Module discovery by exhaustive search for densely connected, co-expressed regions in biomolecular interaction networks

Recep Colak[1], Flavia Moser[1], Jeffrey Shih-Chieh Chu[2], Alexander Schönhuth[3,*], Nansheng Chen[2,*] and Martin Ester[1,*,†]

[1] School of Computing Science
[2] Department of Molecular Biology and Biochemistry
Simon Fraser University
8888 University Drive
Burnaby, BC, V5A 1S6, Canada

[3] Laboratory for Mathematical and Computational Biology
Department of Mathematics
University of California at Berkeley
748 Evans Hall # 3840
Berkeley, CA, 94720-3840, USA

Corresponding author email: ester@cs.sfu.ca

†Corresponding author
*Shared last authorship

May 2, 2010

Abstract

Background: Computational prediction of functionally related groups of genes (*functional modules*) from large-scale data is an important issue in computational biology. Gene expression experiments and interaction networks are well studied large-scale data sources, available for many not yet exhaustively annotated organisms. As has been well-established, when analyzing these two data sources jointly, modules are often reflected by highly interconnected (*dense*) regions in the interaction networks whose participating genes are co-expressed. However, the computational tractability of the associated search problem had remained unclear and methods by which to exhaustively search for such constellations had not been presented.

Methodology/Principal Findings: We provide an algorithmic framework, referred to as *Densely Connected Biclustering (DECOB)*, by which the aforementioned search problem becomes tractable. To benchmark the predictive power inherent to the approach, we computed all co-expressed, dense regions in physical protein and genetic interaction networks from human and yeast. We then reduced our output by an automatized filtering procedure which yields smaller collections of modules which are comparable to those of prior related, state-of-the-art approaches. As a result, we perform favorably in a fair benchmarking competition which adheres to standard criteria. We also point out the advantages of an exhaustive module search, by using the unreduced output to more quickly perform *GO term related* function prediction tasks. We point out the advantages of our exhaustive output by predicting functional relationships using two real case examples.

Conclusion/Significance: We demonstrate that to compute all densely connected and co-expressed regions in interaction networks is an approach to module discovery of ~~great~~ considerable value. Beyond confirming the well settled hypothesis that such co-expressed, densely connected interaction network regions reflect functional modules, we open up novel computational ways to comprehensively analyze the modular organization of an organism based on prevalent and largely available large-scale datasets.

Availability: ~~The core algorithm and the~~ Software and data sets are available at ~~. The postprocessing software is independent of the core technology and is available on request.~~ `http://www.sfu.ca/~ester/software/DECOB.zip`.

1 Author Summary

Computational prediction of functionally related groups of genes (functional modules) based on conveniently available large-scale data in only sparsely annotated organisms has remained an issue of utmost relevance in the post-genomic era. Interaction networks and gene expression experiments are sources of large-scale data which have been widely studied and are well understood. It has been largely supported that modules are often reflected by densely interconnected subnetworks where participating genes are co-expressed. However, the computational tractability of the corresponding computational search problem had remained unclear. In this paper, we describe an algorithmic framework which renders this problem tractable. We then exhaustively search for densely interconnected regions in combinations of protein-protein and genetic interaction networks where genes are co-expressed in both human and yeast. The great benefits of our approach are documented by outperforming related state-of-the-art approaches in a standard benchmarking competition as well as by supporting function prediction tasks which could not be performed as rapidly before.

2 Introduction

On the cellular level, life is driven by chemical compounds acting in concert, in response to internal and external signals. The ultimate goal of investigating the underlying complex molecular patterns is to draw detailed maps of cellular mechanisms, such as metabolic pathways, and their interplay. However, the challenges behind a comprehensive computational and experimental exploration of these mechanisms seem to be daunting, due to the tremendous complexity of living organisms.

The modularity paradigm (1) is a ~~decisive~~ clue to computationally overcome the inherent difficulties in a first important step. This paradigm states that functional subunits of the cellular maps are encoded as *modules*, i.e. groups of functionally related genes. As a most relevant example of practical interest, knowledge about a module facilitates to assign functions to not yet annotated genes modularly associated with fully annotated functional "collaborators". Therefore, the design of biologically sound as well as computationally tractable models for inferring modules has been at the core of functional genomics throughout the post-genomic era.

When searching for modules, approaches that integrate several types of data promise to be superior. Well-known general aspects which support combined analyses are increased robustness w.r.t. the ubiquitous noise in large-scale data, the global correlation between the '*omic*' data types (22; 23) and that single data types provide only partial information. In particular, when jointly analyzing gene expression and interaction data one should consider that:

- Many cellular processes cannot be monitored by studying gene expression alone. For example, several cell-cycle related protein complexes in Yeast contain predominantly housekeeping gene products such

that the functional coherence of the genes of these complexes is not visible on the transcriptional level (24). However, when defined appropriately, co-expressed groups of genes tend to reliably reflect functional modules.

- Subgroups of genes, inferred by screening interaction network data, exhibit quite the opposite behavior. While many more cellular functionalities are reflected by connected subnetworks, the likelihood that a connected subnetwork reflects a functional module is comparatively low. This is due to the fact that interaction networks only provide static pictures of the cell such that the edges in a connected subgraph might not be simultaneously present.

- Based on these two insights we hypothesized that to combine a rather strict network metric (here: *dense* connectivity) with a more relaxed gene expression metric (our definition of co-expression is little restrictive) may result in an excellent, while at the same time computationally manageable definition of a functional module.

In the meantime, a variety of reliable and sound approaches to module discovery has been provided to the related communities. However, some open questions had remained. In particular, none of the established approaches which integrate both network and gene expression data fully resolves the following issues. Note that there are non-integrated approaches which address the issues from below.

- They only provide heuristic solutions to the biologically well-motivated (e.g. (17; 18; 19; 20; 21)), albeit computationally hard problem of searching for densely connected biclusters (in the sense of densely interconnected regions in interaction networks whose participating genes are co-expressed under sufficiently many cellular conditions). Note that *density*, in addition to connectivity, is a recurring theme in approaches based on network data alone (see (9) for a summary).

- They tend to partition the datasets. However, some overlap among the modules is desirable since genes can participate in several, sometimes substantially different, functional contexts.

- While large collections of modules are usually of no immediate practical use, they can be flexibly transformed into smaller outputs of particular interest since they cover the maximum amount of functionalities that can be inferred from the underlying datasets. None of the prior approaches outlines such strategies since they do not find large collections in the first place.

- Gene expression modules tend to reliably reflect functional modules in terms of GO term enrichment, but they do not cover many functionalities since many functionalities do not show on the mRNA level. Network modules show the opposite effect—they achieve good coverage of functionalities since an interaction network usually covers all genes independent of tissue, condition etc. However, network modules often are false positives precisely due to that one cannot ensure that two interactions are active under the same conditions. Combined approaches aim at yielding balanced combinations of enrichment and coverage. However, approaches yielding both enrichment which is on par with methods based on gene expression data alone and coverage comparable with approaches based on network data alone had not been presented yet.

2.1 Approach

The major purpose of this study was to outline ways to exhaustively search for densely connected biclusters in biomolecular network and gene expression data and to elucidate the advantages of such an approach

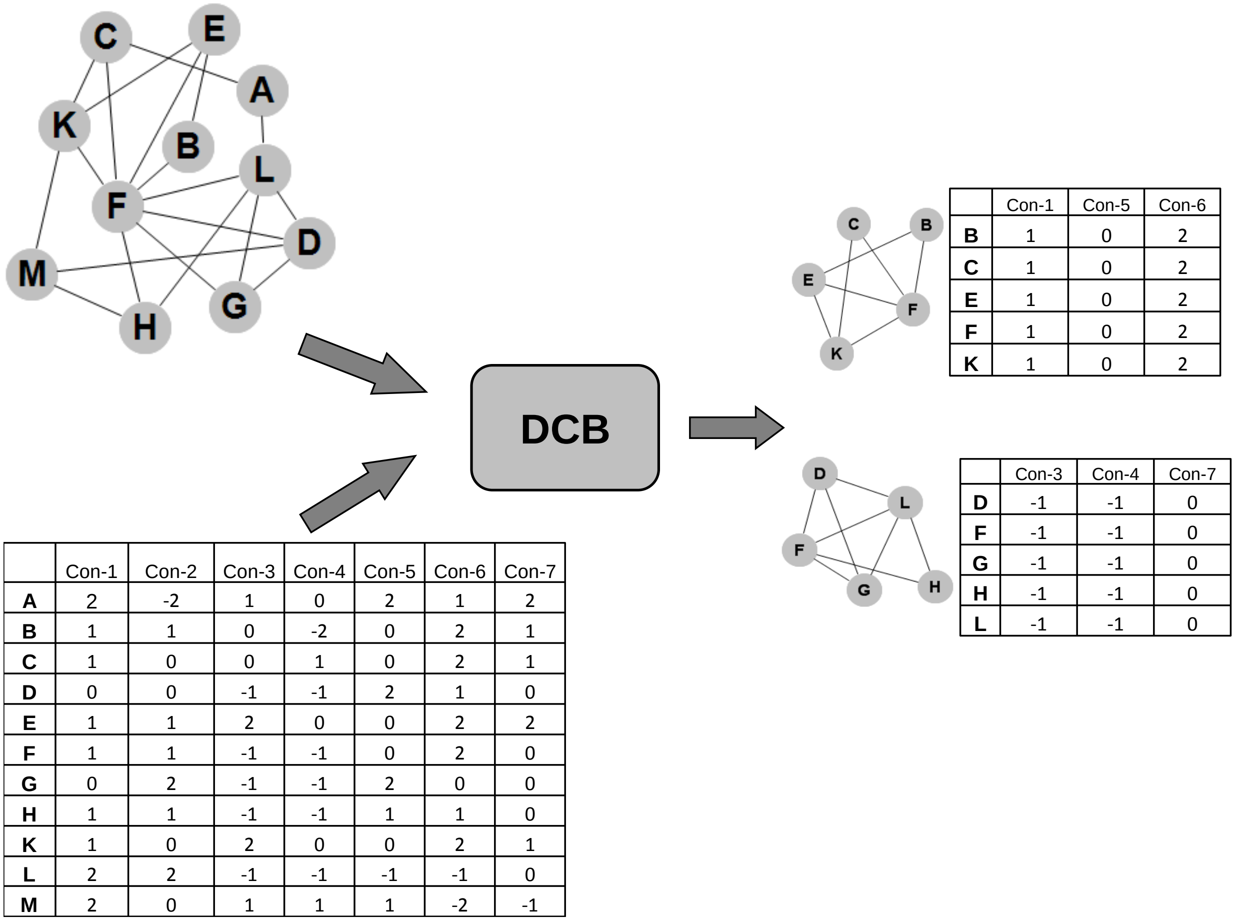

	Con-1	Con-2	Con-3	Con-4	Con-5	Con-6	Con-7
**A**	2	-2	1	0	2	1	2
**B**	1	1	0	-2	0	2	1
**C**	1	0	0	1	0	2	1
**D**	0	0	-1	-1	2	1	0
**E**	1	1	2	0	0	2	2
**F**	1	1	-1	-1	0	2	0
**G**	0	2	-1	-1	2	0	0
**H**	1	1	-1	-1	1	1	0
**K**	1	0	2	0	0	2	1
**L**	2	2	-1	-1	-1	-1	0
**M**	2	0	1	1	1	-2	-1

	Con-1	Con-5	Con-6
**B**	1	0	2
**C**	1	0	2
**E**	1	0	2
**F**	1	0	2
**K**	1	0	2

	Con-3	Con-4	Con-7
**D**	-1	-1	0
**F**	-1	-1	0
**G**	-1	-1	0
**H**	-1	-1	0
**L**	-1	-1	0

Figure 1: Core problem. Groups of genes (B, C, E, F, K) and (D, F, G, H, L) form densely connected biclusters as they form connected subnetworks which contain relatively many edges and genes are co-expressed in sufficiently many cellular conditions.

in the light of the four points from above. We do this by employing a search strategy which was recently presented to the data mining community (58) and tayloring it to the particular requirements when performing functional module discovery. As an illustration see Figure 1. An exhaustive search for maximal densely connected biclusters among the genes $(A, B, C, D, E, F, G, H, K, L, M)$ results in two subgroups: (B, C, E, F, K) and (D, F, G, H, L) both of which are connected and contain at least 7 of the possible $\binom{5}{2} = 10$ edges in the interaction subnetwork (which translates to density ≥ 0.7). These two groups of genes also form biclusters since all of the genes are co-expressed in at least 3 conditions (Con-1, Con-5, Con-6 for (B, C, E, F, K) and Con-3, Con-4, Con-7 for (D, F, G, H, L)) where, in the following, the definition of co-expression will be a more relaxed one. See the Methods section for a formal introduction of the related theory.

The basic idea behind the strategy is to examine all subnetworks of the interaction network for forming a densely connected bicluster but those which can be neglected based on a theorem which was presented in (58) and the theoretical clue is to observe that this renders the computational search problem tractable when screening interaction in combination with gene expression data. The search proceeds in a breadth-first

fashion which translates to first screening all subnetworks of size 2 and proceeding with subnetworks of size n when having enumerated those of size $n-1$. Based on the theorem from (58), we can neglect subnetworks of size n whenever all subnetworks of size $n-1$ contained in the subnetwork of size n are not densely connected biclusters. The theorem ensures that we will not miss a densely connected bicluster. See the subsequent subsection 3.1 for a runtime analysis which shows that our method has reasonable runtimes on the real-world instances considered.

After subsequent application of a novel merging and a novel statistical ranking procedure, we obtain a collection of modules of great quality where modules possibly overlap. The quality of our modules is documented by performing highly favorably in a benchmarking competition. Most importantly, our approach is the only one to achieve top-ranked enrichment and top-ranked coverage simultaneously. Furthermore, we can demonstrate that the overlap among the modules can help to discover different functions of the same gene supported by that a gene may participate in different modules reflecting different functionalities. Last but not least, we show that the comprehensiveness of our output can be used to conveniently perform *function specific* module discovery which will be addressed in subsection 3.5.

2.2 Related Work

Non-Integrative Approaches In large-scale gene expression data, the definition of a module is usually that of a group of co-expressed genes. Several approaches have demonstrated that co-expression significantly increases the likelihood for a group of genes to have similar function (see e.g. (2; 3; 4) for seminal papers). In the meantime, a large variety of inference and clustering algorithms have been presented, often specializing in more specific problem domains. A class of methods that is related to ours are biclustering algorithms. Since the definition of a bicluster is that of a cluster of both genes and cellular conditions, this class of algorithms is particularly suitable when it comes to simultaneously analyzing gene expression data resulting from experiments referring to various different cellular conditions (5; 6). Here, SAMBA (7) proved to be a superior approach in a recent comparative study (8).

Network-based methods for function prediction have been comprehensively reviewed (9). Various network-clustering algorithms and related approaches have been presented since the availability of large-scale network data (e.g. (10; 11; 12; 13), see also the citations in (9)). In an independent comparative study (14), MCL, a Markov chain based method (15; 16) performed most favorably on the suggested benchmarking datasets. Apart from the fact that modules are reflected by connected subgraphs in interaction networks, it is well-established that they usually are also *dense* in terms of above-average edge content. This applies in particular for protein-protein interaction networks (e.g. (20; 21)) since the physical interaction of two gene products is vital for the two genes to commonly exert function. Beyond being applicable for physical interaction networks, this definition also applies for genetic interaction networks . While the correlation of genetic interaction subnetwork patterns with functional entities has not yet been fully explained, a densely connected region in a genetic interaction network usually gives rise to a module. Note, however, that there are exceptions, such as bridge genetic interactions that exist between pathways as compared to within pathway interactions . These cases do not necessarily form a denseregion in a genetic interaction network. In summary, finding densely connected regions in genetic interaction networks alone should yield that the modules are quite trustworthy while not necessarily all modules are discovered. It comes as no surprise that it is common to many of the network-based methods to identify modules according to definitions whose criteria are seemingly correlated (while the exact extent of those correlations usually is not entirely clear) to that of densely connected subgraphs. Despite the successes of the above approaches based on single data types, algorithms that integrate several types of data promise to be superior. Well-known general aspects which support combined analyses are increased robustness w.r.t. the ubiquitous noise in

large-scale data, the global correlation between the '*omic*' data types and that single data types provide only partial information. In particular, when jointly analyzing gene expression and interaction dataone should consider that: Many cellular processes cannot be monitored by studying gene expression alone. For example, several cell-cycle related protein complexes in Yeast contain predominantly housekeeping gene products such that the functional coherence of the genes of these complexes is not visible on the transcriptional level . However, when defined appropriately, co-expressed groups of genes tend to reliably reflect functional modules Note that (21) is the only approach which tries to exhaustively mine for densely connected subnetworks. However, they can only prove to find all dense, but not necessarily connected subnetworks. As a consequence, the devised search strategy can provably miss certain densely connected constellations. Moreover, they do not address how to integrate gene expression data. Subgroups of genes, inferred by screening interaction network data, exhibit quite the opposite behavior. While many more cellular functionalities are reflected by connected subnetworks, the likelihood that a connected subnetwork reflects a functional module is comparatively low. This is due to the fact that interaction networks only provide static pictures of the cell such that the edges in a connected subgraph might not be simultaneously present. Genetic interaction data and physical protein-protein interaction data are often complementary . For example, this was made use of for understanding gene interaction modules in C. elegans early embryogenesis as well as LIN-12-Notch signalling and the actin cytoskeleton pathways . Therefore, combining those two data types can be advantageous.

In general, it is well-established that when combining interaction network and gene expression data, modules are often reflected by *densely connected biclusters*, that is, dense and connected regions in the interaction networks where the participating

Integrated Approaches A recurring theme in earlier approaches is to infer modules as connected subnetworks where genes are co-expressedunder a sufficient number of cellular conditions . However, the tractability of the corresponding computational search problem was largely doubted, due to its hardness in terms of computational complexity.. In the two seminal approaches, Ideker et al. (25) find connected subnetworks which yield a high score measured in P-values obtained from gene expression experiments whereas Hanisch et al. (26) define distance functions, based on both expression and network information, which are subsequently integrated into standard clustering procedures. Segal et al. (27) provided probabilistic graphical models with which to perform combined analysis of interaction network and gene expression data, thereby establishing the first unifying statistical approach to the issue.In the

In the meantime it has become common to the integrated methods to aim at inferring modules as *densely connected* regions in interaction networks that is regions which are not only connected but also contain a high amount of edges, certainly inspired by the successes of approaches based on network data alone which made use of this idea. In fact, it is well-established that when combining interaction network and gene expression data, modules are often reflected by *densely connected biclusters*, that is, dense and connected regions in the interaction networks where the participating genes are co-expressed under a sufficient number of cellular conditions (20). However, the tractability of the corresponding computational search problem had never been demonstrated and all of the previous approaches present related heuristics.

In the most recent approach, Ulitsky and Shamir (28) compute connected subnetworks which, according to a statistical hypothesis test, are significantly co-expressed. Ulitsky and Shamir (28) also report that they outperform prior state-of-the-art approaches in terms of GO term enrichment and coverage (for definitions see Results section below). The modules inferred in (28) are relatively dense (see tables 1, 2) which can be taken as an additional indicator of that functional modules are associated with densely connected, co-expressed subnetworks. Note as well that (28) employ a heuristic, the "heaviest-subnet algorithm" which

computes densely connected interaction subnetworks to be used as seeds in the subsequent main algorithm. However, their method does not explicitly address this issuesolve the problem of exhaustively searching all such subnetwork patterns. See the supplementary materials for a more detailed description. Note also that there are recent approaches addressing how to reliably make use of confidence-scored interaction networks (e.g. (29)). In the following, we do not compare with such methods since confidence scores require a substantial amount of annotations to be trustworthy. Therefore, such approaches refer to a different, though related, problem domain. Here, we would like to focus on module discovery approaches which do not intrinsically rely on annotations. The progress in the recent past has been quite remarkable and biologists have been provided with a variety of reliable and sound approaches to module discovery. However, there are still some disadvantages which are common to all of the existing methods. In particular, none of the established combinedapproaches fully resolves the following issues:

They do not directly address the biologically well-motivated (e.g.), albeit computationally hard problem of searching for densely connected biclusters (in the sense of densely interconnected regions in interaction networks whose participating genes are co-expressed under sufficiently many cellular conditions). They tend to partition the datasets. However, some overlap among the modules is quite desirable since genes can participate in several, sometimes substantially different, functional contexts. While large collections of modules are usually of no immediate practical use, they can be flexibly transformed into smaller outputs of particular interest since they cover the maximum amount of functionalities that can be inferred from the underlying datasets. None of the prior approaches outlines such strategies since they do not find large collections in the first place. On one hand, modules based on restrictive assumptions on the coherence of gene expression patterns tend to reliably reflect functional modules in terms of GO term enrichment, but they do not cover many functionalities. On the other hand, modules predominantly based on network data achieve superior coverage of functionalities while not being similarly enriched. See the discussion above for an explanation. Combined approaches tend to yield combinations of enrichment and coverage which are superior in terms of balanced combinations. However, approaches yielding both high quality enrichment (comparable with modules from gene expression alone) and good coverage (comparable to that of modules inferred from network data alone) have been still missing. Most approaches focused on how to promisingly process

Interaction Data Beyond being applicable for physical interaction networks, the definition of dense connectivity also makes sense when screening genetic interaction networks for modules (17). While the correlation of genetic interaction subnetwork patterns with functional entities has not yet been fully explained, a densely connected region in a genetic interaction network usually gives rise to a module. Note, however, that there are exceptions, such as bridge genetic interactions that exist between pathways as compared to within pathway interactions (19). These cases do not necessarily form a dense region in a genetic interaction network. In summary, finding densely connected regions in genetic interaction networks alone should yield that the modules are quite trustworthy while not necessarily all modules are discovered. Note also that genetic interaction data and physical protein-protein interaction data in combination with gene expression data. Approaches which addressed how to promisingly make use of the complementarity of protein-protein and genetic interaction data, in combination with gene expression data have not yet been evaluated.Since interaction networks tend to be rather sparse, a combined analysis of protein-protein and genetic interaction data should further improve on coverage of functionalities. are often complementary (19). For example, this was made use of for understanding gene interaction modules in C. elegans early embryogenesis (18) as well as LIN-12-Notch signalling and the actin cytoskeleton pathways (66). Therefore, combining those two data types can be advantageous.

The motivation of this study was to provide a unifying algorithmic framework by which these issues could be addressed. In the following, we will introduce such a framework based on computing the entirety of densely connected , possibly overlapping, biclusters in an organism (see Methods section) and we will demonstrate the benefits of this approach when comparing it with state-of-the-art methods for module discovery and/or computational function prediction (see Results section). We will discuss our findings conclusively in the Discussion section.

3 Results

In the following, we As a starting point we computed all densely connected biclusters in both Yeast and Human according to definition 5.1 in the Methods section. We then distinguish between two methods which emerge from our algorithmic frameworkresult from further processing the exhaustive set of all these densely connected biclusters. The output of the first, called *DECOB (DEnsely COnnected Biclustering)* , consists of all densely connected biclusters according to definition 5.1 in the Methods section where, subsequently , is obtained by subsequently merging biclusters which share a large dense coreare merged in a postprocessing procedure which has a biological motivation (see Methods section and . This is motivated by that biclusters which substantially overlap do not differ much in terms of their functional interpretation. See also (31))for a related discussion. We refer to this output the set of biclusters where substantially overlapping modules heve been merged as *DECOB* modules in the following. See subsection 5.3 and subsection For further details, see subsections 5.3 and 5.4 for a fully detailed description.

The output of the second method, *DECOBRA (DECOB RAnked)*, has been specifically tailored to serve the purposes of a fair benchmarking procedure. It consists of the *DECOB* modules which remain after having applied an automatized ranking-based filtering procedure to the *DECOB* modules which results in a reduced number of modules, referred to as *DECOBRA* modules. See subsection 5.6 for a full description of the ranking-based filtering procedure.

We computed all densely connected biclusters in both Human and Yeast, based on standard gene expression and protein-protein / genetic interaction network datasets (see subsection 5.1 for a more detailed explanation). In order to demonstrate the benefits of our approach we then computed all *DECOB* modules and, by means of the above mentioned filtering procedure, the *DECOBRA* modules. We then performed

1. a standard benchmarking competition (see subsection 3.2) for which we suggest *DECOBRA* as a fair competitor and

2. evaluated the (unreduced) set of *DECOB* modules when employed for specific function prediction tasks (see subsection 3.5) which require large and comprehensive sets of high-quality modules as a basis.

3.1 Tractability: Runtime Analysis

In order to give evidence that our approach achieves reasonable runtimes on biological problem instances of interest we tested our software on the Yeast dataset for varying choices of $\alpha, \theta_{exp}, \theta_d$ (for exact definitions of those parameters which quantify subnetwork density and co-expression, see the Methods section). Thereby, we left two of the parameters $\alpha, \theta_{exp}, \theta_d$ fixed at $\alpha = 0.65, \theta_{exp} = 1.25, \theta_d = 140$ according to what was found a biologically motivated choice in Yeast and varied the third, remaining parameter. See Figure 2 for corresponding statistics. As one can see the combination of $\alpha = 0.65, \theta_{exp} = 1.25, \theta_d = 140$ resulted in about 15 seconds runtime to process the Yeast dataset. Changing θ_{exp} and θ_d resulted in changes in

runtime on the order of (up to 100) seconds (Figure 2, top and middle). Changing α makes the most significants effects as was to be expected due to the exponential increase in search space size (Figure 2, bottom). As mentioned above we recall that the DCB constraints are only loose anti-monotone for $\alpha \geq 0.5$ which requires to invoke additional subroutines in order to find all densely connected biclusters for choices of $\alpha < 0.5$ (see (63; 58) for details). However, even for the most problematic choices of $\alpha \leq 0.4$ the runtime is only on the order of a few minutes beyond that such choices are biologically not necessarily well motivated in module discovery.

3.2 Standard Function Prediction Benchmarking

The general outline of the following competition has been adopted from approved, earlier studies (28). In the following, we refer to a group of potentially functionally related genes as inferred by any of the methodologies under consideration as a *(functional) module*. To directly compare the predictive power of the complete output of *DECOB* with those of the benchmarking competitors would be inappropriate since the complete output of *DECOB* is one order of magnitude larger than the outputs of the other methods in terms of inferred modules. The idea behind approaches yielding rather small outputs is to provide the experimenter with only a small collection of modules of utmost quality. Since the technologies behind the approaches of the competitors exclusively address this idea, a direct comparison of our collection with theirs would be misleading. Therefore, we have developed an automatized ranking procedure which, when applied to the complete output of *DECOB* yields an output that can be compared with the outputs of the other methods in a fair comparison. See subsection 5.6 in the Methods section for further details. As mentioned above, we refer to the combined application of *DECOB* and ranking-based filtering procedure which yields the sort of output which can be incorporated into a meaningful benchmarking procedure as *DECOBRA* in the following. In general, the output of *DECOBRA* can be used for common function prediction tasks in the sense of the earlier approaches.

As benchmarking competitors, we chose four related publicly available, state-of-the-art algorithms as well as a randomized baseline method. The two integrated methods we chose are *CO-Clustering (COC)* which is a seminal approach on the topic (26) whereas *MATISSE* (28) can be considered to have set the current standards since it performed quite favorably with respect to the methods under consideration. We also have benchmarked against two methods that operate on single data types (either interaction network or gene expression data). While *MCL* (15) operates on only interaction network data, *SAMBA* (7) only operates on gene expression data (note that *SAMBA* can in theory also be used to integrate other types of data, but has not been thoroughly evaluated for such tasks. Both methods can be considered to have established the gold standard on the types of data under consideration here. The randomized baseline method (*Rand. Conn.*) randomly sampled connected PPI networks (we obtained empirical module size distributions from the output sets of all algorithmic approaches and sampled connected networks according to that size distribution). In the supplementary materials we provide a more detailed description of the algorithmic technologies which underlie the approaches of the competitors. Thereby, we put particular emphasis on the issues under special consideration here, such as overlap and density. For all algorithms, we used the recommended parameter settings if applicable.

Module Assessment We measured several GO-based quantities to assess module quality. The most important definitions of quantities have been adopted from earlier studies (28). For all calculations, we used the high-throughput version of the GoMiner tool (30).

Basic Statistics (# genes, # modules, average module size (AMS), Density (DY)). These numbers provide insights about the number of genes covered by the inferred modules as well as the number of modules,

Table 1: Benchmarking Competition Yeast

	Basic Statistics				Quality Measures		
**Benchmarking Competitors**	#Gen.	#Mod.	AMS	DY	ER	COV	IC
Samba	876	135	25.96	.02	90 (**2**)	11	20
MCL	693	95	7.29	.44	~~89~~88	30 (**1**)	~~21~~ 33 (**2**)
Matisse	360	17	21.17	.31	95 (**1**)	6	17
COC	986	103	9.57	.06	72	19	16
Rand. Conn.	737	134	16.87	.27	~~85~~84	23	4
DECOBRA	576	354	9.33	.41	95 (**1**)	29 (**2**)	41 (**1**)
**Additional Methods**	#Gen.	#Mod.	AMS	DY	ER	COV	IC
DECOBRA Top-100	226	100	13.14	.33	100	5	18
DECOBRA Top-200	388	200	11.49	.35	97	16	31
DECOB	576	2276	9.33	.39	~~94~~93	46	55

their average size and their average density. These basic statistics may also assist in choosing convenient methods according to practical considerations. Average density (see Def. 5.1) reveals how density is related to module quality.

Enrichment (ER) is a standard measure and possibly the most important one. It can be interpreted as the probability that an inferred module is a set of functionally related genes. It is computed as the percentage of modules found that are enriched with at least one GO term of level[1] 7 or higher (meaning 8,9,...), as suggested in (28)) with P-values, that were corrected for multiple hypothesis testing, below a threshold of 0.01.

Coverage (COV) is another standard measure (see (28)). It is the number of GO terms that were enriched in any of the inferred modules divided by the number of all GO terms associated with the interaction network and gene expression datasets under consideration.

Lastly, as genes can be associated with GO terms reflecting different functionalities which indicates their participation in several functional contexts, we suggest **Individual Coverage (IC)** as a quantity which measures how well the functionalities of the individual genes are covered. IC is the probability that, given a gene and one of its associated GO terms, the GO term is enriched in one of the inferred modules containing that gene. More formally, if N is the number of genes, let $F(G)$ be the number of terms associated with gene G that are enriched in inferred modules that contain the gene and $T(G)$ be the total number of terms associated with that gene then

$$\textbf{IC} = \frac{1}{N} \sum_{G} \frac{F(G)}{T(G)}$$

where the sum ranges over all genes G. It measures how many of the functional contexts of a gene are covered by the output. To put it into another context it measures how well a method can identify multiple functions of a gene. Therefore, it comes as no surprise that methods which yield non-overlapping modules have rather low IC (see tables 1 and 2).

In general, it is quite hard to provide a truly fair benchmarking competition, due to different numbers of

[1] In this context, level means the length of the shortest directed path from the node associated with the most generic GO term to the target GO term based on the child-parent relations as induced by the topological organization of GO

Table 2: Benchmarking Competition Human

	Basic Statistics				Quality Measures		
**Benchmarking Competitors**	#Gen.	#Mod.	AMS	DY	ER	COV	IC
Samba	1709	129	48.94	.01	95 (**1**)	13	12
MCL	1863	312	5.94	.35	81	58	27 (**2**)
Matisse	1364	76	17.94	.30	~~94~~ 93 (**2**)	25	18
COC	3558	271	13.12	.01	79	44	7
Rand. Conn.	1921	406	10.18	.35	~~89~~88	61 (**1**)	3
DECOBRA	1358	758	6.52	.46	95 (**1**)	60 (**2**)	37 (**1**)
**Additional Methods**	#Gen.	#Mod.	AMS	DY	ER	COV	IC
DECOBRA Top-100	347	100	7.12	.44	97	15	10
DECOBRA Top-200	553	200	7.08	.44	95	26	~~16~~15
DECOB	1358	5979	7.12	.45	~~98~~97	64	51

covered genes and modules of the competitors. We suggest *DECOBRA* as a competitor since its numbers of covered genes are roughly on an equal level with the other methods while the number of modules does not differ in terms of orders of magnitude. The output of *DECOBRA* results from application of a ranking based filtering procedure to the complete set of densely, connected and co-expressed interaction subnetworks (*DECOB* modules). See subsection 5.6 for a detailed description of *DECOBRA*. We recall that the complete set of *DECOB* modules, without subsequent application of the ranking procedure results in substantially larger number of genes and modules (see the DECOB row in the tables 1, 2). We would finally like to point out that the design of strategies for comparison of clustering / module discovery methods which yield overlapping outputs is an active area of research (e.g. (14)). As mentioned above, we opted to have each method roughly the same amount of genes covered which is fair with respect to have everyone a "best bet" on the functions of the same amount of genes. The subsequent results can generally be interpreted as the difference of having less, but usually larger, non-overlapping modules (competitors) in contrast to our approach which yields more, but smaller and overlapping modules.

Yeast Table 1 displays the statistics as defined in subsection 3.2 that were achieved by the comparison partners on both yeast and human datasets (see subsection 5.1). In each column of the table, we have highlighted the methods that perform best or second best (position in parentheses).

In *Yeast*, *DECOBRA* is first in ER and IC and second in COV. *MCL* wins COV, one obvious reason being that it assigns each gene to a module, thereby achieving high coverage rates. However, *MCL*'s performs rather poorly (relative to the baseline established by *Rand. Conn.*) in ER, which is considered to be the measure of individual module quality. This is likely due to yielding subnetworks as modules where edges are not simultaneously present since it does not consider gene expression data and confirms the intuitive idea about the limitations of static network data when it comes to function prediction. Nevertheless, we recall that *MCL* proved to perform very favorably among the methods that consider network data alone (14) in an independent comparative study (14). At any rate, it is interesting to observe the high density of the modules inferred by *MCL*. We would also like to mention the high enrichment value of *Matisse*. Also here, the relatively high density of the output modules (although this is not explicitly part of its underlying module definition) might come as no surprise. Clearly, a general explanation for *Matisse*'s module quality is that it

is an integrated approach. Note that the only method which achieves both top-ranked enrichment as well as top-ranked coverage is *DECOBRA*.

Human Table 2 displays the statistics defined in subsection 3.2 that were achieved by the comparison partners on the human datasets (see subsection 5.1). In *Human*, *DECOBRA* finishes shared first in ER, second in COV and first in IC. Note first that the random baseline method *Rand. Conn.* wins COV. This is a clue to the questionability of COV as the only measure of how well functionalities are covered by a module discovery program and points out that further, more appropriate quality measures are required. Note, for example, that *Rand. Conn.* performs suboptimal, if not poorly in the non-standard measures IC which we had suggested for further evaluation since they convey meaning of obvious interest in function prediction. In this context, note also that MCL performs slightly worse in COV, but superior (being second best) in IC. This further supports that coverage of functionalities is hard to assess and that novel ways for doing so are needed.

The high ER achieved by *SAMBA* is remarkable (sharing the first position with *DECOBRA*) which confirms that biclustering is a highly valuable approach when considering gene expression data alone. Moreover, it confirms that co-expression, if appropriately defined, is a strong indicator of functional relationships. However, *SAMBA*'s COV and IC are rather poor reflecting that not every functional relationship becomes visible in terms of co-expression *SAMBA* modules. Note that *DECOBRA* employs a rather relaxed definition of co-expression whose predictive power comes from combining it with the retrieval of interaction relationships. Both *MCL* and *COC* achieve relatively good coverage values, again an obvious reason being that they assign each gene to a module. However, for both of them, ER is even worse than that of the baseline method (*Rand. Conn.*). Last but not least, note that *Matisse* achieves top-rated ER values also on the human dataset. In conclusion, note that *DECOBRA* is the only method to achieve both top-ranked ER and COV among all competitors.

3.3 DECOB

For general interest, we also display results for the full set of *DECOB* modules, computed as per the algorithmic procedure of subsection 5.4. We are also showing the results for the top 100 and top 200 *DECOB* modules that result from stopping the filtering procedure after having filtered out 100 resp. 200 *DECOB* modules (*DECOBRA Top-100* resp. *DECOBRA Top-200*). See the lower parts of the tables 1 and 2 for corresponding statistics. *DECOB*'s high ER, in particular in Human (ER=98) is quite remarkable since it can be related to that, in Human, 98% of all densely connected, co-expressed subnetworks are GO term enriched which underscores the applicability of the widely believed idea that such constellations reflect cellular functional entities. Furthermore, note that all of the top 100 *DECOB* modules in Yeast are enriched. Last but not least note that *DECOB* achieves overall best values in COV and IC in both Human and Yeast. In accordance with the definitions of COV and IC, these are also strong clues to the benefits of *DECOB* when performing more specific function prediction tasks where large amounts of high quality modules are needed as a ground set. This will be described in the subsequent subsection 3.5.

3.4 Advantages of Overlapping Modules

A first clue to the benefits of allowing for overlap among the inferred modules are the good IC values of *DECOBRA* (we recall the definition of IC as the probability that a gene / GO term combination is reflected by a module containing the gene and being enriched relative to the GO term), in both Yeast and Human.

Table 3: Statistics on overlapping module pairs supporting different functionalities (OMPSDF)

Organism	Samba	MCL	Matisse	COC	DECOBRA
Yeast	4	0	0	0	1264
Human	208	0	0	0	194

This can be explained by that modules can be overlapping allowing for that genes can be involved in several modules referring to different functional contexts. Note that the unreduced output of *DECOB* achieves even better IC values which gives evidence of the benefits of an exhaustive approach in this respect.

In Table 3 we have further evaluated how well the individual methods perform with respect to revelation of the different functionalities of the genes. It is intuitively obvious that overlap is a crucial necessity to properly reflect the different functionalities of a single gene. To further examine this we have counted all module pairs (M_1, M_2) (OMPSDF = Overlapping Module Pairs Supporting Different Functionalities) in Table 3 such that

1. The intersection of M_1 and M_2 is not empty.
2. M_1 and M_2 do not share (in terms of enrichment) a GO term at level 3. This translates to that they reflect different cellular core processes.
3. Among the genes which are shared by M_1 and M_2 there is a gene which is annotated with two GO terms of level 3 or below $(4, 5, 6, ...)$ where one of the terms is enriched in M_1 (hence not in M_2, since M_1 and M_2 do not share such terms) and the other term is enriched in M_2 (hence not in M_1). This means that the gene supports two functionalities which are essentially different.

The number of such pairs of modules produced by the different methods are shown in Table 3. As expected, none of the methods which partition the datasets (MCL, Matisse, COC), in particular none of the prior combined ones (Matisse, COC) do infer such module pairs. The only method apart from DECOBRA which outputs such configurations is Samba, which operates on gene expression data alone. The differences between the numbers in Yeast and Human are due to the peculiarities of the gene expression datasets under consideration. Note that the 1264 pairs reported correspond to 2% of the $\binom{354}{2}$ possible pairs where 354 is the number of modules output by DECOBRA (see Table 1) which means that in Yeast 2% of the DECOBRA module pairs support the desirable idea of finding constellations where genes interact in different cellular functional contexts. Only DECOBRA reports substantial amounts of such overlapping configurations.

3.5 Advantage of Exhaustive Searches

In the following, we would like to demonstrate the advantages of an exhaustive module search by describing an experimental scenario of practical interest. The idea is to provide one or several functionalities of specific interest and then to select all modules from the output of a module discovery method which are enriched with functionalities under consideration. This aims at integrating partial knowledge in terms of functionalities in order to more specifically predict gene and protein function in rather sparsely annotated organisms. The resulting collections of modules should reflect functionalities which are related to the functionality specified. Since it would be desirable to be able to add or combine functionalities interactively and not to have to recompute module collections upon modification of specification of functionalities, an advantageous workflow of such studies would be to

1. first compute a large collection of high quality modules and
2. then to interactively select collections of specific interest by simple filtering procedures.

Clearly, in order to support such an advantageous workflow, the initial collection should be both comprehensive and rich in terms of functionalities covered and reliable in terms of module quality. Revisiting the statistics of the tables 1 and 2 reveals that the exhaustive collection of *DECOB* modules meets these criteria since it achieves superior module quality and superior coverage of functionalities, unlike the approaches with reduced outputs. We would like to mention that none of these approaches have been designed to support such workflows in the first place (see the supplementary materials for a detailed description of their methodologies) and that it would be interesting to see whether their module definitions can be used for such exhaustive searches when attuned accordingly. Here, we compare the specific *DECOB* collections with the specific collections that result from filtering the output of the benchmarking competitors for modules which are enriched with a GO term of particular interest in order to demonstrate that reduced, unspecific collections are not appropriate. Note that a general clue to the reliability of the *DECOB* output for GO term specific modules is not only provided by its excellent ER and COV, but also its superior IC values (see the DECOB row in tables 1 and 2). The IC value in particular gives evidence that more functionalities per gene will be covered in general. Hence our specific collections will give rise to comprehensive predictions of very high reliability.

We display a detailed analysis of the collection of modules which resulted from two GO term specific function prediction performances, one in Human and one in Yeast. In Yeast, we focused on GO term "GO:0006333, chromatin assembly" whereas in Human we focused on GO term "GO:0060070, Wnt receptor signaling pathway through beta-catenin". Our choice of GO terms was motivated by our own research interests. While "Wnt receptor signaling pathway through beta-catenine" plays an important role in development, "chromatin assembly" is critical for regulating gene expression. We collected modules from all methods under consideration, by selecting only those which were enriched with one of the two GO terms. Subsequently, we analyzed these collections.

Yeast: GO:0006333, Chromatin Assembly *DECOB* provided us with 56 modules which were enriched with genes associated with chromatin assembly. These 56 modules contained on average 11 genes and had an average overlap of 25%.

As a first point, our analysis revealed interesting interrelationships in the *DECOB* modules. Note that we can compute a ranking of the modules, as is described in subsection 5.6. We found that the module which was top-ranked among the 56 modules carried particularly interesting, potentially novel, information about chromatin assembly. See the ~~left part in~~ top of Figure 3 for a picture.

In more detail, this module presents 13 members that function in chromatin structural modification. 5 members encode histone subunits: HTB2 (YBL002W), HTA1 (YDR225W), HTA2 (YBL003C), HHF1 (YBR009C), and HHF2 (YNL030W). We recall that histones are core proteins that DNA wraps around to form nucleosomes. Histones, especially the tails, can be modified to form euchromatin or heterochromatin structures which are commonly associated with transcriptionally active region and transcriptionally silent region, respectively. HST3 (YOR025W) is an example of deacetylase that removes acetyl groups from histones (specifically H3K56) to promote formation of heterochromatin (32; 33). HST3 works in concert with RTT107 (YHR154W) and other proteins to establish transcriptional silencing in locus such as HMR, HML,

and telomeres (34). ASF1 (YJL115W) also facilitates gene silencing by promoting nucleosome assembly by chromatin assembly factor I (CAF-I) (35; 36). This notion is supported by yeast strains with mutation in ASF1 show defects in heterochromatic gene silencing (37). ASF1 binds acetylated form of histones and stimulates nucleosome assembly in an HIR and POL30 (YBR088C) dependent manner (36). The mechanism and interaction between ASF1, HIR proteins, and POL30 is still unclear. Heterochromatin assembly, kinetochore formation, and chromosome segregation is a tightly linked process. SWI6 in S. pombe functions in gene silencing, kinetochore assembly, and microtubule attachment to kinetochores (38; 39). Similarly, CAF-I and HIR proteins in S. cerevisae, which are important for heterochromatin assembly, also function in kinetochore assembly (40). Other proteins that have a role in this coordinated process include SMC5 and SMC6 (YLR383W). SMC5-SMC6 complex are localized to centromeres and are crucial for proper chromosome segregation both in S. pombe (41; 42) and in S. cerevisae (43; 44). It is therefore no surprise that kinesins CIN8 (YEL061C) and CIK1 (YMR198W) are also members of the module where they are crucial for structural integrity of mitotic spindle during mitosis when chromosomes segregate (45). CIN8 and CIK1 are readily degraded by CDH1. CLB2 (YPR119W) activates a mitotic kinase CDC28 to inhibit CDH1 to allow accumulation of CIN8 and CIK1 (46). Taken together, all 13 members of this module are reasonably grouped. Our study here suggests histone and histone modification proteins work in a concerted effort with kinetochore proteins and kinesins during mitosis. This has been shown to some extent with CAF-I and HIR proteins (40). According to our module, ASF1, which functions together with CAF-I and HIR, may also function in kinetochore formation and chromosome segregation during mitosis.

An analysis of the modules of the other methods revealed that *DECOB* is the only method that makes such prediction. In general, the modules generated by DECOB are not found by any of the other methods. Moreover, the modules from other programs with GO-term enrichment in chromatin assembly/disassembly show limited overlap with the *DECOB* modules.

Conversely, the other methods predict genes to be associated with chromatin assembly or related processes which cannot be found in any of the *DECOB* modules. The *COC* modules contain 18 genes where 4 are Histone genes, 10 are ribosomal proteins, and the rest are membrane associated or membrane transport protein. The relationship between the members of this module in terms of chromatin assembly/disassembly does not become obvious. Similarly, the *Matisse* modules do not present obvious relationships in terms of chromatin assembly/disassembly. Here, out of 24 genes in total, 5 are histone genes, 9 are ribosomal proteins, and some genes involved in RNA processing and amino acid degradation. Apart from *DECOB*, the *MCL* module presents the most plausible predictive quality. It consists of polymerase, topoisomerase, and DNA repair genes. However, *MCL* only generates one module, consisting of 6 genes. The histone genes are clearly missing in this module. Lastly, one *SAMBA* module (cluster 72) shows high overlap (among all enriched *SAMBA* modules) with the top-ranked *DECOB* module we analyzed. However, while being of high overlap, it also has genes not directly related to chromatin assembly/disassembly such as genes involved in nuclear export, mRNA localization, Golgi membrane protein, and zinc transporter protein.

Last but not least, the *DECOB* modules are generally better enriched in terms of p-values.

Human: GO:0060070, Wnt Receptor Signaling Pathway through Beta-Catenin *DECOB* delivered 17 modules which were enriched with genes associated with GO term GO:0060070. These 17 modules contained 8 genes on average and had an average overlap of 13%. In the following, we focus on analyzing the *DECOB* module which was most significantly enriched since it carried particularly interesting interrelationships. We will comment on its contents in more detail in the following, before turning our attention to the modules of the other methods. See the ~~right part~~ bottom of Figure 3 for a picture. Note already now that

only *COC* and *MCL* returned modules which were enriched with GO:0060070.

The selected *DECOB* module consists of 7 genes: COBRA1, CTNNB1, ERBP, ESR1, GSK3B, MNAT1, and SMAD2. CTNNB1 and GSK3B are known members in β-catenin signaling. CTNNB1 (also known as β-catenin) is a key component in Wnt signaling that is able to translocate to the nucleus to modify many transcription factors such as lymphoid enhancer factor (LEF) (47) and FOXO transcription factors (48). GSK3B regulates CTNNB1 level by phosphorylating CTNNB1 for degradation (49). SMAD2 is a member of the TGF-β signaling pathway. The remaining 4 genes (COBRA1, ERBP, ESR1, and MNAT1) are part of the estrogen receptor pathway. ESR1 (estrogen receptor 1) is a ligand-activated transcription factor that binds to the estrogen-response element (ERE) while ERBP (estrogen receptor binding protein) binds to and enhances ESR1 activity. ESR1 activity is regulated by a number of factors. COBRA1 interacts with ESR1 and is also able to inhibit ESR1 target gene activation upon estrogen stimulation (51). Similarly, MNAT1 also interacts and translocates with ESR1 upon estrogen activation (52). It is suggested that ESR1 activity may be influenced by MNAT1 via chromatin remodeling (52). It is only recently that we begin to see some evidence suggesting the convergence of the estrogen receptor pathway and Wnt signaling pathway. Kouzmenko et al. showed in Drosophila that ERβ (ESR1) functionally interacts with β-catenin and that β-catenin can be recruited to EREs (53). Mendez et al. similarly showed that GSK3 positively regulate estrogen receptor activity in N2a cells by enhancing transcription of target genes (54). Having this in view, the *DECOB* module under consideration presents some interesting predictions of potential novel interactions between Wnt signaling pathway and estrogen receptor pathway.

Another interesting feature was to observe that overlap, ESR1 was found to participate in *DECOB* modules, different from the one under consideration here, which were enriched with the GO term "Estrogen receptor pathway", but not with the GO term under consideration here ("Wnt signalling pathway through β-catenine"). This is a concrete example of the benefits of overlapping modules, which, in this example, share ESR1 as a member, but reflect different functionalities.

None of the modules of the other methods make such predictions. Aside from *DECOB*, *COC* and *MCL* are the only two programs that return a module which is enriched with GO term GO:0060070. None of the above methods generates a module from the *DECOB* output. The only *COC* module contains 8 genes. While some of them are for DNA repair (MRE11A, POLI, RUVBL2), one is associated with microtubule regulation (MAPRE1). It is not obvious at the point if any cross talk occurs between Wnt signaling pathway and DNA repair. *MCL* on the other hand yields a 17 gene module that is highly enriched in cell adhesion and junction proteins such as cadherins (4 genes), catenin (3 genes), desmosomes (2 genes), and their associated proteins (4 genes). Some of these genes, like β-catenin, have a membrane associated form functioning in cell-cell contact and a cytoplasmic form to function in signaling pathway but the idea that all adhesion molecules also play a role in Wnt signaling is currently not supported by the literature. At any rate, there are similar *DECOB* modules, predicting similar contexts as the *MCL* module. Note, however, that the *DECOB* modules show higher significance in GO terms relating to cell-cell junction than Wnt signaling.

In summary, our analysis reveals that the GO-term specific collection of *DECOB* modules possesses the better predictive power, since it reveals well-known, relevant and predicts plausible, interesting relationships that other methods miss.

4 Discussion

In the Introduction, we had outlined that despite the great advances in the area of functional module discovery which were made in the post-genomic era, a few issues whose overcoming promised further potentially significant improvements had remained unresolved.

First, the tractability of the computational problem to exhaustively search for *densely connected biclusters*, that is, dense and connected regions in interaction networks where genes are sufficiently co-expressed ~~was largely doubted~~had remained an unresolved issue. However, the idea that densely connected biclusters reliably reflect functional modules was widely supported and well-established (e.g. (17; 18; 20; 21)). Beyond the cited evidence, it is interesting to notice that MCL which operates on network data only and performed quite favorably in a comparative study (14), employs a definition which is akin to that of densely connected regions in the interaction networks (see the supplementary materials for a detailed description of MCL). However, none of the approaches which operate on both interaction network and gene expression data, explicitly addresses this objective. Also, while there is evidence in the literature that dense connectivity gives rise to reliable modules in genetic interaction networks (17; 19), none of these approaches were evaluated on such data.

Second, prior combined approaches tend to partition the datasets, thereby establishing one-to-one correspondences between genes and functionalities although it is well-known that genes can participate in several functional contexts. Note that methods which operate only on gene expression data can infer overlapping modules (55; 56; 57) which underscores the benefits of this idea.

Third, there were no approaches which generated large, comprehensive collections of high-quality modules resulting from exhaustive screens of the modular organization of organisms. The idea behind such exhaustive searches is to subsequently tailor the resulting large collections to more specific needs, by means of fast filtering strategies. Apart from convenience in such annotation-specific module discovery tasks, exhaustive collections may also provide a global picture of the modular organization of an organism.

In this paper, we have presented an algorithmic framework with which to resolve the outlined issues. The framework is centered around the problem of exhaustively searching for *densely connected biclusters*. Thereby, it was crucial to observe that the so called *DCB constraints* which give rise to densely connected biclusters are *loose antimonotone* (see the Methods section and, for a full description of the mathematical details, also (58)). The property of loose antimonotonicity can be naturally incorporated into an algorithmic strategy by which to efficiently search for densely connected biclusters. Thanks to our strategy, the resulting collections of densely connected biclusters are overlapping and no densely connected bicluster is missed by our procedure which results in a large, comprehensive collection of high-quality modules.

In order to validate the usefulness and to demonstrate the benefits of our approach, we have tested two module discovery methods, *DECOB* and *DECOBRA* which emerge from our framework. While the output of *DECOB* results from merging densely connected biclusters which share a significantly large overlapping core, the output of *DECOBRA* results from significantly reducing the output of *DECOB* according to a ranking-based filtering procedure. This serves the purposes of a fair competition since its output is comparable to the outputs of the prior approaches in terms of numbers of modules and of genes covered. We then employed *DECOBRA* in a standard benchmarking procedure. The comprehensive output of *DECOB* was employed to predict functional relationships of particular interest using two real case examples. For this purpose, the output of *DECOB* was filtered according to the particular interests as specified by two GO terms.

In the benchmarking competition, *DECOBRA* proved to be superior over the prior, state-of-the-art approaches under consideration. While this is good evidence of that densely connected biclusters indeed

reliably reflect functional modules, we observed some further interesting phenomena:

1. Our randomized baseline method, which operates on interaction network data only, achieved respectable enrichment (ER) values (see tables 1 and 2), which underscores that connectivity is a valuable concept when screening interaction network data. This also shows that achieving enrichment up to 90% does not require elevated levels of sophistication. However, the fact that it also achieves respectable coverage values is quite disturbing and casts certain doubts on enrichment and coverage as the only measures to assess the performance of module discovery programs. As a first attempt to mend these deficiencies we introduced IC which reflects how many functionalities per gene are covered on average. It is interesting to observe that the randomized baseline method achieves only poor values here, whereas the sophisticated module finder MCL, which also operates on interaction network data only, achieves superior values in these novel categories while being at most on par with the randomized baseline method in the standard values.

2. We observed that MCL achieved good coverage values (COV and IC) while achieving only relatively low (below 90%) enrichment (ER). This reflects that, on one hand, quite a substantial percentage of functional contexts is reflected by interaction data. However, on the other hand, still a significant amount of dense and connected regions do not reflect modules, likely due to the fact that the underlying combinations of edges are not simultaneously present within cellular contexts. In other words, interaction network data is *static*.

3. SAMBA, which operates only on gene expression data, achieves good (even superior enrichment values in the human data set) without achieving good overall coverage. This reflects that coherent expression patterns indicate modular arrangements when co-expression is appropriately modeled. However, not all modular arrangements become visible at the transcriptional level which is a well-known fact (24).

4. The essence of the previous points is that a good idea for module discovery approaches is to employ the comprehensive predictive power of interaction network data while using gene expression as a control element. Thereby, one must be aware of that state-of-the-art definitions of co-expression (such as the one of SAMBA) could rule out too many network patterns, which would again result in low coverage values. Note that the definition of co-expression of our approach is a rather relaxed one. To our understanding, the combination of network and expression criteria as per our approach explains both the superior enrichment and superior coverage.

Note finally that, independent of the fairness issues (*DECOB*'s output is larger by one order of magnitude than the outputs of the benchmarking competitors), *DECOB* achieves the best values in all benchmark measures. We filtered the output of *DECOB* by specifying GO terms of interest and studied the resulting real case examples of module collections. By doing so, we aimed at demonstrating that the large output of *DECOB* can be employed to more conveniently tackle more specific function prediction tasks. A thorough analysis of the specific collection of *DECOB* modules and the (much smaller) collections of the prior approaches revealed that the *DECOB* modules possess the greatest predictive power, since it reveals well-known, relevant and predicts plausible, interesting relationships that other methods miss. It would be interesting to see how related approaches perform when being tailored to address such tasks. However, it remains unclear to what extent the comparison partners considered here (see the supplementary materials for detailed descriptions of the approaches) can be modified to support such tasks.

In summary, we have provided evidence of the substantial benefits of our module discovery framework when it comes to resolving the issues outlined in the Introduction. Future work will be concerned with adapting our methodology to confidence-scored interaction data, which has received considerable attention in the recent past. Moreover, we are planning to explore the applicability of gene co-expression constraints which are different from that of Definition 5.1. For example, there usually is a negative correlation between genetic interacting genes belonging to alterative pathways. As such, order preserving submatrix analysis is a promising direction as it can handle both positive and negative correlation (6). Last but not least, mining modules with density thresholds that are related to module size in the style of (70) should be beneficial. Note that in (70), the determination of significance thresholds for subgraph size dependent density is incorporated into a mining algorithm which, in contrast to our approach, partitions the networks into a fragmentary collection of subgraphs hence outputs an incomplete, non-overlapping collection of modules. Combining a subgraph size significance analysis with an exhaustive search for densely connected biclusters should yield further improvements in module discovery.

5 Methods

5.1 Data

Yeast We extracted the interaction network, containing both PPI and GI interactions from multiple publicly available datasets from the BioGRID database (59). Gene expression data was given by the yeast compendium dataset (60). It reports fold changes of experiment against control in as many as 300 cDNA experiments. We discarded genes whose ratios were to be found in a 1.5 times variance interval around the mean over all conditions, hence nowhere exhibited significant expression levels. This amounted to 1043 differentially expressed genes with 2664 interactions in the resulting network.

Human Again, the PPI/GI network was downloaded from the BioGRID database (59). For the gene expression data, we used the comprehensive human tissue expression dataset (61), which lists fold changes over 115 cDNA experiments across 35 different tissue types. In order to account for activity, we only retained variably expressed genes which were with at least 2-fold ratio variation from the mean in at least two samples, as suggested by the authors of (61). As a result, the human dataset contained 3628 genes connected by 8924 interactions in the respective network.

5.2 General Strategy

On a high level, our method consists of the following steps:

1. Infer the entirety of all densely interconnected subgraphs whose genes are co-expressed (definitions see subsection 5.3, *DECOB* algorithm, see subsection 5.4).
2.
   - In order to provide specific collections of modules, specify the functionality of interest and filter the (comprehensive) output accordingly (results see subsection 3.5)
   - In order to obtain a small and reliable collection of modules which is independent of choices of GO terms, we apply a ranking procedure that ranks the modules according to density and coherence in expression. We then select modules using these rankings as a guide, *without* that numbers of modules have to be specified beforehand (*DECOBRA*, see subsection 5.6).

5.3 Densely Connected Biclustering: Problem Definition and Properties

In order to formally introduce our problem definition, we will employ the following terminology.

A **profile network** is defined as an undirected graph $G = (V, E, F)$ consisting of a node (gene) set V, an edge set E and a **profile function**

$$\begin{array}{rccc} F: & V & \to & \mathbb{R}^K \\ & v & \mapsto & (F_1(v), ..., F_K(v)). \end{array}$$

F assigns a fold change expression profile to each node of an interaction network. For $K' \subset \{1, ..., K\}$, we will refer to $\mathbb{R}^{K'}$, the projection of $\mathbb{R}^K$ onto the dimensions specified by K', as a **profile subspace**. We are interested in the following three properties of an induced subnetwork $G' = G[V'] = (V', E', F)$: co-expression, density and connectedness, which are summarized in the following definition.

~~Example of DCBs where $\alpha = 0.7, \theta_d = 3, \theta_{exp} = 0.$~~

Definition 5.1 (Densely connected Biclustering). *Let $G' = G[V'] = (V', E', F)$ be an induced subnetwork of a profile network $G = (V, E, F)$.*

1. *G' is **co-expressed** wrt. (with respect to) θ_d, θ_{exp} if there is a profile subspace $\mathbb{R}^{K'}$, $|K'| \geq \theta_d$ such that for all $k \in K'$*

$$\max_{v,v' \in V'} |F_k(v) - F_k(v')| \leq \theta_{exp}.$$

   This translates to that the expression levels of genes v' of the subgraph $G[V']$ do not differ by more than θ_{exp} under the at least θ_d many cellular conditions indexed by K'. Note that a set of correspondingly co-expressed genes can be viewed as a **bicluster** *of genes and conditions in the sense of the usual definition of a bicluster.*

2. *The **density** of $d(G')$ of G' is defined as the ratio of the number of edges in G' over the number of possible edges in G',*

$$d(G') = \frac{|E'|}{\binom{|V'|}{2}} = \frac{2|E'|}{|V'|(|V'| - 1)}.$$

   *We say that G' is α-**dense** if*

$$d(G') \geq \alpha.$$

3. *G' is **connected** if there exists a path in G' between any pair of nodes in V'.*

4. *G' is called a **densely connected bicluster (DCB)** or, equivalently, satisfies the DCB* **constraints** *wrt. $\alpha, \theta_d, \theta_{exp}$ if G' is connected, co-expressed wrt. θ_{exp} and θ_d and α-dense. A DCB G' is* **maximal** *if it is not a proper subgraph of another densely connected bicluster.*

See Figure 1 for an example of a densely connected bicluster.

Biological Instances: In the instances of profile networks $G = (V, E, F)$ considered, V is a set of genes/gene products and edges E correspond to both PPI and GI interactions. F can be identified with fold change expression profiles of the genes. Accordingly, a DCB will be a set of genes that are co-expressed within a θ_{exp} fold-change neighborhood of each other across at least θ_d experimental conditions and whose associated nodes form a densely connected interaction subnetwork (see Figure 1).

Densely Connected Biclustering (DCB) Problem:

Input: Profile network $G = (V, E, F)$, density threshold α, homogeneity threshold θ_{exp} and minimum number of dimensions θ_d.
Output: All maximal DCBs of G satisfying the DCB constraint specified by α, θ_{exp} and θ_d.

The DCB problem is $\mathcal{NP}$-hard. Its decision version is $\mathcal{NP}$-complete, shown by a simple reduction from the max-clique problem (62). As a straightforward observation note that naive approaches to the DCB problem would require an exhaustive enumeration of all 2^N subnetworks of G, which is infeasible in general (here, N will be on the order of the number of genes in an organisms hence on the order of several thousands). In case of PPI/GI networks, tractability is provided based on the following observation.

Definition 5.2 (Loose Anti-Monotonicity)**.** *A constraint is called* ***loose anti-monotone*** *if for each network G of size n that satisfies the constraint, one can find at least one induced subnetwork $G' \subset G$ of size $n-1$ satisfying the constraint.*

The crucial observation for rendering the search problem tractable is that the DCB constraints are loose anti-monotone if $\alpha \geq 0.5$. Below we provide a proof sketch for this to hold. Detailed definitions and fully elaborated proofs can be found in (63) and (58).

Proof Sketch: Clearly, the co-expression constraint holds for all induced subnetworks of size $n-1$ of a DCB G' of size n. We can therefore restrict our attention to dense connectivity. To obtain a DCB of size $n-1$ of G', one tries to remove the node (and with it its edges) whose degree is smallest. We will be done if the resulting network is still connected. If the network is disrupted into two sets of nodes, then the smaller one of the components, including the disrupting node, contains at most half of the nodes of the original network. This translates to that the degree of these nodes, divided by the number of possible incident edges ($= n-1$) is at most 0.5. Therefore, some straightforward computations reveal that one can remove all these nodes without violating the density constraint. It remains to observe that removing a node in the smaller component that is farthest away (in terms of shortest paths) from the disrupting node will not disrupt the network. $\diamond$

We would finally like to point out that for $1/3 \leq \alpha < 0.5$ the DCB constraints are not loose anti-monotone. By means of further theorems and, based on them, additional subroutines that follow the core routine from below, we would have been able to infer all α-dense DCBs also for $1/3 \leq \alpha < 0.5$ (see (63; 58) for details).

In order to have an appropriate choice of α we examined the densities of the Yeast protein complexes and pathways. See subsection 5.5 for a more detailed description.

Related Work A most recent approach whose theoretical framework supports inference of all dense, but not necessarily connected subgraphs in interaction networks (without particularly addressing gene expression) is (21). While they employ their methodology to only search for connected, dense subnetworks, the algorithmic strategy does not guarantee to do this exhaustively and one can show that they miss certain dense and connected subnetworks. The idea of mining for densely interconnected subgraphs was also successfully applied to co-expression networks where edges connect genes when they are significantly co-expressed across a range of different cellular conditions. In this case, several specifically adapted heuristics were devised to tackle the corresponding search problems (64; 65).

5.4 DECOB Algorithm

The core strategy of $DECOB$ is to narrow down the huge search space consisting of the 2^N (where N is the number of the nodes of the network, i.e. the number of genes in an organism) subnetworks of the original network by means of the loose anti-monotonicity of the $DECOB$ constraint. See Figure 4 for an example. In a preprocessing step, we remove edges whose nodes refer to genes that are not sufficiently co-expressed, that is edges between genes whose expression profiles do not meet the co-expression constraint, as such edges cannot participate in any $DECOB$ (note that the co-expression constraint, taken by itself, is strongly anti-monotone which means that none of the children of such gene pairs can meet the co-expression constraint). Then we conceptually organize all connected subnetworks in a hierarchical structure (formally a lattice) where a subnetwork is a child of another one if it can be obtained by adding exactly one gene (and the corresponding edges) that is connected to the parent subnetwork. Note that a child is larger than its parent which may be a bit counterintuitive.

We then traverse this structure top-down, in a breadth-first search. This means that subnetworks of size n are only checked upon having produced all $DECOB$s of size $n-1$. The point is that when it comes to examining subnetworks of size n, we can restrict ourselves to checking children of $DECOB$s of size $n-1$, as the loose anti-monotonicity of the $DECOB$ constraints guarantees that every $DECOB$ of size n necessarily has a $DECOB$ of size $n-1$ as a parent. For example, in Figure 4 only children of the $DECOB$s A-B-D and B-C-D are examined further whereas other subnetworks (e.g. A-B-C-E) are not checked as they have no $DECOB$ as a parent. If a $DECOB$ cannot be expanded by a node (i.e. it is *maximal*) it is returned as an output. The only maximal $DECOB$ in Figure 4 is A-B-C-D.

In order to both increase and adequately evaluate the biological quality of our modules we subsequently employ a refinement procedure. Its biological motivation is that, in biomolecular networks, functional subunits often consist of a *dense* core in combination with genes which are "attached" to the core (e.g. (31)). The following refinement procedure will merge DCBs when they overlap to a high degree. Ensembles of genes resulting from this merging procedure reflect such "core-attachment" constellations. In addition to such motivation, missing data is another issue that we address by the refinement procedure.

We iteratively merge pairs of DCBs if they overlap in at least 75% of their members as well as in at least 80% of their associated co-expression subspaces (referring to the gene expression conditions under which they are sufficiently co-expressed). This is because currently available PPI/GI networks are far from being complete as well as that gene expression experiments contain a high amount of noise. These issues result in significant amounts of modules that are split up into fractions. The refinement step alleviates this problem by relaxing the density and co-expression constraints in such cases. Note that the refinement procedure implies that, despite our choice of a density threshold of 0.65 (see below), the density of the inferred modules can be lower than 0.65.

Throughout the paper, we refer to the modules which result from merging DCBs as described above as either $DECOB$s or $DECOB$ modules, depending on the context.

5.5 Choice of Parameters

In order to choose α appropriately we examined the average density of the Yeast protein complexes and the pathways as downloaded from the SGD database (66). See Figure 1 on page 5 of the supplementary materials for corresponding statistics. While the mean density of those complexes was found to be 0.79, we found that the average density of the annotated modules was reduced upon combination of the two datasets and subsequent removal of nodes which referred to genes with missing gene expression data. Therefore, we chose $\alpha = 0.65$ as a biologically well motivated density threshold and, based on the underlying biological

inspiration and the good results we obtain, we suggest this choice of parameter as a default value. Note that the density of our modules can become lower than 0.65 upon treatment in the postprocessing step.

Similarly, based on the distributions of the number of co-expressed dimensions of the annotated modules, we further chose $\theta_{exp} = 1.25$ and $\theta_d = 140$ (out of 300) for the yeast dataset. Contrary to yeast, there is no comprehensive true human module dataset. With regard to the fact that the human expression dataset contains a high amount of missing values ($> 25\%$) which adverts to a high amount of noise, we used more relaxed thresholds ($\theta_{exp} = 1.4$, $\theta_d = 10$ (out of 115)).

5.6 ~~Scalability of the Algorithm~~

~~Runtimes on the Yeast dataset for varying density thresholds α for fixed $\theta_{exp} = 1.25, \theta_d = 140$ (bottom), varying dimensionality thresholds θ_d for fixed $\alpha = 0.65, \theta_{exp} = 1.25$ (top left), varying co-expression thresholds θ_{exp} for fixed $\alpha = 0.65, \theta_d = 140$ where homogeneity refers to co-expression (top right)~~

~~In order to examine the scalability of our approach we tested our software on the Yeast dataset for varying choices of $\alpha, \theta_{exp}, \theta_d$. Thereby, we left two of the parameters $\alpha, \theta_{exp}, \theta_d$ fixed at $\alpha = 0.65, \theta_{exp} = 1.25, \theta_d = 140$ according to what was found a biologically motivated choice in Yeast and varied the third, remaining parameter. See Figure 2 for corresponding statistics. As one can see the combination of $\alpha = 0.65, \theta_{exp} = 1.25, \theta_d = 140$ resulted in about 15 seconds runtime to process the Yeast dataset. Changing θ_{exp} and θ_d resulted in changes in runtime on the order of (up to 100) seconds (Figure 2, top left and top right). Changing α makes the most significants effects as was to be expected due to the exponential increase in search space size (Figure 2, bottom). As mentioned above we recall that the DCB constraints are only loose anti-monotone for $\alpha \geq 0.5$ which requires to invoke additional subroutines in order to find all densely connected biclusters for choices of $\alpha < 0.5$ (see for details). However, even for the most problematic choices of $\alpha \leq 0.4$ the runtime is only on the order of a few minutes beyond that such choices are biologically not necessarily well motivated in module discovery.~~

5.6 DECOBRA Algorithm

In order to provide a competitor which meets the purposes of the standard benchmarking procedure, we have developed a filtering procedure which is based on a ranking of the output of *DECOB*. Thus, the output of *DECOBRA (DEnsely COnnected Biclustering RAnked)* results from subsequent filtering of the output of *DECOB*, as described below.

Ranking: Co-expression Ranking To assess the significance of the co-expression encountered in the output modules, we randomly sampled 2000 connected networks from the instances at hand (see the result section 3). We fitted the resulting statistics on numbers of co-expressed conditions to a truncated normal distribution which provided us with a p-value for the $DECOB$ under consideration.

Ranking: Dense Connectivity Ranking Let N be the number of nodes and K be the number of edges in the complete network under consideration. We interpret the probability that a subnetwork of size n, sampled randomly from the network, has k edges as the corresponding probability of the hypergeometric distribution (which, as a toy description, refers to sampling $\binom{n}{2}$ red balls from an urn with $\binom{N}{2}$ balls K of which are red). We are aware of that counting subgraphs in biomolecular networks and/or respective statistics are areas of active research (e.g. (67; 68; 69)). Note that the hypergeometric distribution is in accordance with the analyses displayed in (70) hence represents a reasonable choice

Overall Ranking and Filtering We ranked the modules according to both co-expression and dense connectivity separately and used the average of the rankings as an overall ranking. This yields a ranked list of the output of DECOB. In order to filter *DECOB's* output accordingly, we traversed the ranking list from top to the bottom and removed all modules whose genes were contained in the modules higher up in the ranking list. The remaining modules are the output of *DECOBRA*. In order to obtain even smaller outputs we suggest to select only the K best ranked modules from the output of *DECOBRA* since this yields both high-quality and non-redundant collections of modules. See also the lower parts of the tables 1 and 2 for module statistics on such smaller collections ($K = 100$ and $K = 200$).

Author Contributions

Conceived and designed the experiments: RC, AS, ME, NC. Performed the experiments: RC. Analyzed the data: JSC, RC, AS, NC. Wrote the paper: AS, RC, FM, JSC, NC. Contributed (theoretical groundwork): RC, FM Other (Designed the method): RC, FM, ME. Other (Implemented the method): RC.

Acknowledgements

Recep Colak was funded by the Canadian Institute for Health Research (CIHR) / Michael Smith Foundation for Health Research (MSFHR) Bioinformatics Training Program for Health Research. Recep Colak, Alexander Schönhuth, Martin Ester and Nansheng Chen are members of the Bioinformatics for Combating Infectious Diseases (BCID) project, which is funded by the Simon Fraser University (SFU) Community Trust Endowment Fund. Alexander Schönhuth was also funded by a postdoctoral stipend from the Pacific Institute for the Mathematical Sciences (PIMS). Martin Ester and Nansheng Chen are supported in part by a National Sciences and Engineering Research Council (NSERC) Discovery grant. Nansheng Chen is also an MSFHR scholar.

Figure Legends

Figure 3: Two real case examples of a Yeast (~~left~~top) and a Human (~~right~~bottom) module as inferred by application of DECOB and further filtering by GO terms of specific interest. The Yeast module on the left was obtained by screening the output of DECOB for modules which are enriched with the GO term "Chromatin Assembly" (GO:0006333). The Human module on the right was obtained by screening the output of DECOB for modules which are enriched with the GO term "Wnt Receptor Signaling Pathway through Beta-Catenin" (GO:0060070).

Figure 1: This figure refers to the definition of a densely connected bicluster (Definition 5.1 in the main text) referring to the parameters $\alpha = 0.7$ (density) and $\theta_d = 3, \theta_{exp} = 0$ (co-expression constraints). The input for the core algorithm is the interaction network of the organism (here, as a toy example, genes A,B,C,D,E,F,G,H,I,J and K) together with gene expression dataset containing (logarithmic) fold changes of genes across a set of experimental conditions (here: the table below the interaction network). On the right, we display the set of densely connected biclusters which refer to the datasets on the left. The densely connected biclusters contain at least $0.7 (= \alpha)$ times the amount of possible edges and its genes are co-expressed in at least 3 different experimental conditions ($\theta_d = 3$) with a difference of at most 0 ($\theta_{exp} = 0$).

Figure 4: Illustration of the $DECOB$ algorithm on a simplified example consisting of six genes and three gene expression conditions. $DECOB$ constraints are specified by: $\alpha = 0.8$ (density), $\theta_{exp} = 0.5$ (maximum difference in expression) and $\theta_d = 2$ (number of expression conditions). The algorithmic strategy is to traverse the lattice of all subnetworks in a breadth-first fashion. Any subnetwork which is not a densely connected biclusters can be discarded due to that every densely connected bicluster necessarily has a densely connected bicluster as a parent (= subnetwork contained in the original one, see definition 5.2 and the discussion below of it). For esthetical reasons, we have omitted B-C-D-E although, as a child of the densely connected bicluster B-C-D, it is also examined. B-C-D-E, just as A-B-D-E will be discarded since it violates the density constraint.
Figure 2: Runtimes of our algorithm for varying, biologically relevant choices of the parameters involved in our framework. The most important observation is that the algorithm is scalable we have runtimes of at most a few minutes for all choices of α (density).

References

[1] Albert, R. 2005. Scale-free networks in cell biology. *J. of Cell Science*, 118, 4947-4957.

[2] Eisen, M.B., Spellman, P.T., Brown, P.O, and Botstein, D. 1998. Cluster analysis and display of genome-wide expression patterns. *Proc. Natl. Acad. Sci.*, 95, 14863-14868.

[3] Tamayo, P., Slonim, D., Mesirov, J., Zhu, Q., Kitareewan, S., Dmitrovsky, E., Lander, E.S., and Golub, T.R. 1999. Interpreting patterns of gene expression with self-organizing maps: Methods and application to hematopoietic differentiation. *Proc. Natl. Acad. Sci.*, 96, 2907-2912.

[4] Tavazoie, S., Hughes, J.D., Campbell, M.J., Cho R.J., and Church, G.M. 1999. Systematic determination of genetic network architecture. *Nature Genetics*, 22, 281-285.

[5] Cheng, Y., and Church, G.M. 2005. Biclustering of expression data. In *Proc. of the Eighth Int. Conference on Intelligent Systems for Molecular Biology*, 93-103.

[6] Ben-Dor, A., Chor, B., Karp, R., and Yakhini, Z. 2003. Discovering local structure in gene expression data: The order-preserving submatrix problem. *J. of Computational Biology*, 10(3-4), 373-384.

[7] Tanay, A., Sharan, R., and Shamir, R. 2002. Discovering statistically significant biclusters in gene expression data. *Bioinformatics*, 18 (Suppl. 1), 136-144.

[8] Prelić, A., Bleuler, S., Zimmermann, P., Wille, A., Brühlmann, P., Gruissem, W., Hennig, L., Thiele, L., and Zitzler, E. 2006. A systematic comparison and evaluation of biclustering methods for gene expression data. *Bioinformatics*, 22(9), 1222-1129.

[9] Sharan, R., Ulitsky, I., and Shamir, R. 2007. Network-based prediction of protein function. *Molecular Systems Biology*, 3:88.

[10] Bader, G.D., and Hogue, C.W. 2003. An automated method for finding molecular complexes in large protein interaction networks. *BMC Bioinformatics*, **4:2**.

[11] Spirin, V., and Mirny, L.A. 2003. Protein complexes and functional modules in molecular networks. *Proc. Natl. Acad. Sci.*, 21, 12123-12126.

[12] King, A.D., Przulj, N., and Jurisica, I. 2004. Protein complex prediction via cost-based clustering. *Bioinformatics*, 20(17), 3013-3020.

[13] Sharan, R., Ideker, T., Kelley, B., Shamir, R., and Karp, R.M. 2005. Identification of protein complexes by comparative analysis of yeast and bacterial protein interaction data. *J. of Computational Biology*, 12, 835-846.

[14] Brohee, S., and van Helden, J. 2006. Evaluation of clustering algorithms for protein-protein interaction networks. *BMC Bioinformatics*, 7:488.

[15] Enright, A.J., Van Dongen, S., and Ouzounis, C.A. 2002. An efficient algorithm for large-scale detection of protein families. *Nucleic Acids Research*, 30, 1575-1584.

[16] Krogan, N.J., *et al.* 2006. Global landscape of protein complexes in the yeast Saccharomyces cerevisiae. *Nature*, 440, 637-643.

[17] Tong, A.H.Y., *et al.* 2004. Global Mapping of the Yeast Genetic Interaction Network. *Science*, 303, 808-813.

[18] Gunsalus, K., Ge, H. et al. 2005. Predictive models of molecular machines involved in Caenorhabditis elegans early embryogenesis. *Nature*, 436, 861-865.

[19] Beyer, A., Bandyopadhyay, S. and Ideker, T., 2007. Integrating physical and genetic maps: from genomes to interaction networks. *Nature Reviews Genetics*, 8, 699-710.

[20] Zhu, X., Gerstein, M., and Snyder, M. 2007. Getting connected: analysis and principles of biological networks. *Genes and Development*, 21, 1010-1024.

[21] Georgii, E., Dietmann, S., Uno, T., Pagel, P. and Tsuda, K. 2009. Enumeration of condition-dependent dense modules in protein interaction networks. *Bioinformatics*, 25:7, 933-940.

[22] Ge, H., Liu, Z., Church, G.M., and Vidal, M. 2001. Correlation between transcriptome and interactome mapping data from Saccharomyces cerevisiae. *Nature Genetics*, 29:4, 482-486.

[23] Grigoriev, A. 2001. A relationship between gene expression and protein interactions on the proteome scale: analysis of the bacteriophage T7 and the yeast Saccharomyces Cerevisiae. *Nucleic Acids Research*, 29:17, 3513-3519.

[24] de Lichtenberg, U., Jensen, L.J., Brunak, S. and Bork, P. 2005. Dynamic complex formation during the yeast cell cycle. *Science*, 307, 724-727.

[25] Ideker, T., Ozier, O., Schwikowski, B., and Siegel, A.F. 2002. Discovering regulatory and signaling circuits in molecular interaction networks. *Bioinformatics*, 18, 233-240.

[26] Hanisch, D., Zien, A., Zimmer, R., and Lengauer, T. 2002. Co-clustering of biological networks and gene expression data. *Bioinformatics*, 18 (Suppl. 1), 145-154.

[27] Segal, E., Wang, H., and Koller, D. 2003. Discovering molecular pathways from protein interaction and gene expression data. *Bioinformatics*, 19 (Suppl. 1), 264-271.

[28] Ulitsky, I., and Shamir, R. 2007. Identification of functional modules using network topology and high-throughput data. *BMC Systems Biology*, 1:8.

[29] I. Ulitsky and R. Shamir, 2009. Identifying functional modules using expression profiles and confidence-scored protein interactions. *Bioinformatics* **25**, 1158-1164.

[30] Zeeberg, R.B., *et al.* 2003. GoMiner: A Resource for Biological Interpretation of Genomic and Proteomic Data. *Genome Biology*, 4:4, R28.

[31] Leung, H.C., Xiang, Q., Yiu, S.M. and Chin, F.Y. 2009. Predicting protein complexes from PPI data: a core-attachment approach. *Journal of Computational Biology* 16(2), 133-144.

[32] Celic, I., et al., 2006. The sirtuins hst3 and Hst4p preserve genome integrity by controlling histone h3 lysine 56 deacetylation. *Curr Biol*, 16(13): p. 1280-9.

[33] Yang, B., A. Miller, and A.L. Kirchmaier, 2008. HST3/HST4-dependent deacetylation of lysine 56 of histone H3 in silent chromatin. *Mol Biol Cell*, 19(11): 4993-5005.

[34] Zappulla, D.C., et al., 2006. Rtt107/Esc4 binds silent chromatin and DNA repair proteins using different BRCT motifs. *BMC Mol Biol*, 7: 40.

[35] Tyler, J.K., et al., 1999. The RCAF complex mediates chromatin assembly during DNA replication and repair. *Nature*, 402(6761): 555-60.

[36] Sharp, J.A., et al., 2001. Yeast histone deposition protein Asf1p requires Hir proteins and PCNA for heterochromatic silencing. *Curr Biol*, 11(7): 463-73.

[37] Singer, M.S., et al., 1998. Identification of high-copy disruptors of telomeric silencing in Saccharomyces cerevisiae. *Genetics*, 150(2): 613-32.

[38] Grewal, S.I. and S. Jia, 2007. Heterochromatin revisited. *Nat Rev Genet*, 8(1): 35-46.

[39] Yamagishi, Y., et al., 2008. Heterochromatin links to centromeric protection by recruiting shugoshin. *Nature*, 455(7210): 251-5.

[40] Sharp, J.A., et al., 2002. Chromatin assembly factor I and Hir proteins contribute to building functional kinetochores in S. cerevisiae. *Genes Dev*, 16(1): 85-100.

[41] Pebernard, S., et al., 2004. Nse1, Nse2, and a novel subunit of the Smc5-Smc6 complex, Nse3, play a crucial role in meiosis. *Mol Biol Cell*, 15(11): 4866-76.

[42] Pebernard, S., et al., 2008. Localization of Smc5/6 to centromeres and telomeres requires heterochromatin and SUMO, respectively. *Embo J*, 27(22): 3011-23.

[43] Torres-Rosell, J., et al., 2005. SMC5 and SMC6 genes are required for the segregation of repetitive chromosome regions. *Nat Cell Biol*, 7(4): 412-9.

[44] Lindroos, H.B., et al., 2006. Chromosomal association of the Smc5/6 complex reveals that it functions in differently regulated pathways. *Mol Cell*, 22(6): 755-67.

[45] Bouck, D.C., A.P. Joglekar, and K.S. Bloom, 2008. Design features of a mitotic spindle: balancing tension and compression at a single microtubule kinetochore interface in budding yeast. *Annu Rev Genet*, 42: 335-59.

[46] Crasta, K., et al., 2008. Inactivation of Cdh1 by synergistic action of Cdk1 and polo kinase is necessary for proper assembly of the mitotic spindle. *Nat Cell Biol*, 10(6): 665-75.

[47] Caspi, M., et al., 2008. Nuclear GSK-3beta inhibits the canonical Wnt signalling pathway in a beta-catenin phosphorylation-independent manner. *Oncogene*, 27: 3546-55.

[48] Essers, M.A., et al., 2005. Functional interaction between beta-catenin and FOXO in oxidative stress signaling. *Science*, 308: 1181-4.

[49] Rubinfeld, B., et al., 1996. Binding of GSK3beta to the APC-beta-catenin complex and regulation of complex assembly. *Science*, 272: 1023-6.

[50] Hoover, L.L. and Kubalak, S.W., 2008. Holding their own: the noncanonical roles of Smad proteins. *Sci Signal*, 1: pe48.

[51] Aiyar, S.E., et al., 2004. Attenuation of estrogen receptor alpha-mediated transcription through estrogen-stimulated recruitment of a negative elongation factor. *Genes Dev*, 18: 2134-46.

[52] Talukder, A.H., et al., 2003. MTA1 interacts with MAT1, a cyclin-dependent kinase-activating kinase complex ring finger factor, and regulates estrogen receptor transactivation functions. *J Biol Chem*, 278: 11676-85.

[53] Kouzmenko, A.P., et al., 2004. Wnt/beta-catenin and estrogen signaling converge in vivo. *J Biol Chem*, 279: 40255-8.

[54] Mendez, P. and Garcia-Segura, L.M., 2006. Phosphatidylinositol 3-kinase and glycogen synthase kinase 3 regulate estrogen receptor-mediated transcription in neuronal cells. *Endocrinology*, 147: 3027-39.

[55] Gasch, A.P., and Eisen, M.B. 2002. Exploring the conditional coregulation of yeast gene expression through fuzzy k-means clustering. *Genome Biology*, 3, RESEARCH0059.

[56] Schliep, A., Steinhoff, C., and Schönhuth, A. 2004. Robust inference of groups in gene expression time-courses using mixtures of HMMs. *Bioinformatics*, 20 (Suppl. 1), i283-i289.

[57] Schliep, A., Steinhoff, C., Costa, I. and Schönhuth, A. 2005. Analyzing gene expression time-courses. *IEEE/ACM Transactions on Computational Biology and Bioinformatics*, 2(3), 179-193.

[58] Moser, F., Colak, R., Rafiey, A. and Ester, M. 2009. Mining cohesive pattern from graphs with feature vectors. *SIAM International Conference on Data Mining (SDM 2009).*

[59] Stark, C., Breitkreutz, B-J., Reguly, T., Boucher, L., Breitkreutz, A., and Tyers, M. 2006. BioGRID: a general repository for interaction datasets. *Nucleid Acid Research*, 34 (Database issue), 535-539.

[60] Hughes, T., *et al.* 2000. Functional Discovery via a Compendium of Expression Profiles. *Cell*, 102, 109-126.

[61] Shyamsundar, R., Kim, Y.H., Higgins, J.P., Montgomery, K., Jorden, M., Sethuraman, A., van de Rijn, M., Botstein, D., Brown, P.O., and Pollack, J.R. 2005. A DNA microarray survey of gene expression in normal human tissues. *Genome Biology*, 6:R22.

[62] Karp, R.M. 1972. Reducibility Among Combinatorial Problems, 85-103. *In* Miller, R.E., and Thatcher, J.W., eds., *Complexity of Computer Computations*. Plenum Press, New York.

[63] Colak, R. 2008. *MSc Thesis*, School Of Computing Science, Simon Fraser University, ftp://fas.sfu.ca/pub/cs/theses/2008/RecepColakMSc.pdf.

[64] Hu, H. *et al*. (2005) Mining Coherent Dense Subgraphs Across Massive Biological Networks for Functional Discovery. *Bioinformatics*, **21**(Suppl.1), i213-i221.

[65] Yan, X. *et al*. (2007) A graph-based approach to systematically reconstruct human transcriptional regulatory modules. *Bioinformatics*, **23(13)**, i577-i586.

[66] Botstein, D., Chervitz, S.A., and Cherry, J.M. 1997. Yeast as a model organism. *Science*, 277, 1259-1260.

[67] Milo, R., Shen-Orr, S., Itzkovski, S., Kashtan, N., Chklovskii, D., and Alon, U. 2002. Network motifs: simple building blocks of complex networks. *Science* 298, 824-827.

[68] Grochow, J., and Kellis, M. 2008. Network motif discovery using subgraph enumeration and symmetry-breaking. *Proc. RECOMB 2008*, 92.

[69] Alon, N., Dao, P., Hajirasouliha, I., Hormozdiari, F., and Sahinalp, S.C. 2008. Biomolecular network motif counting and discovery by color coding. *Bioinformatics*, 24 (Proceedings of the ISMB 2008), i241-i249.

[70] Koyuturk, M., Szpankowski, W. and Grama, A. 2007. Assessing significance of connectivity and conservation in protein interaction networks. *Journal of Computational Biology*, 14(6), 747-764.

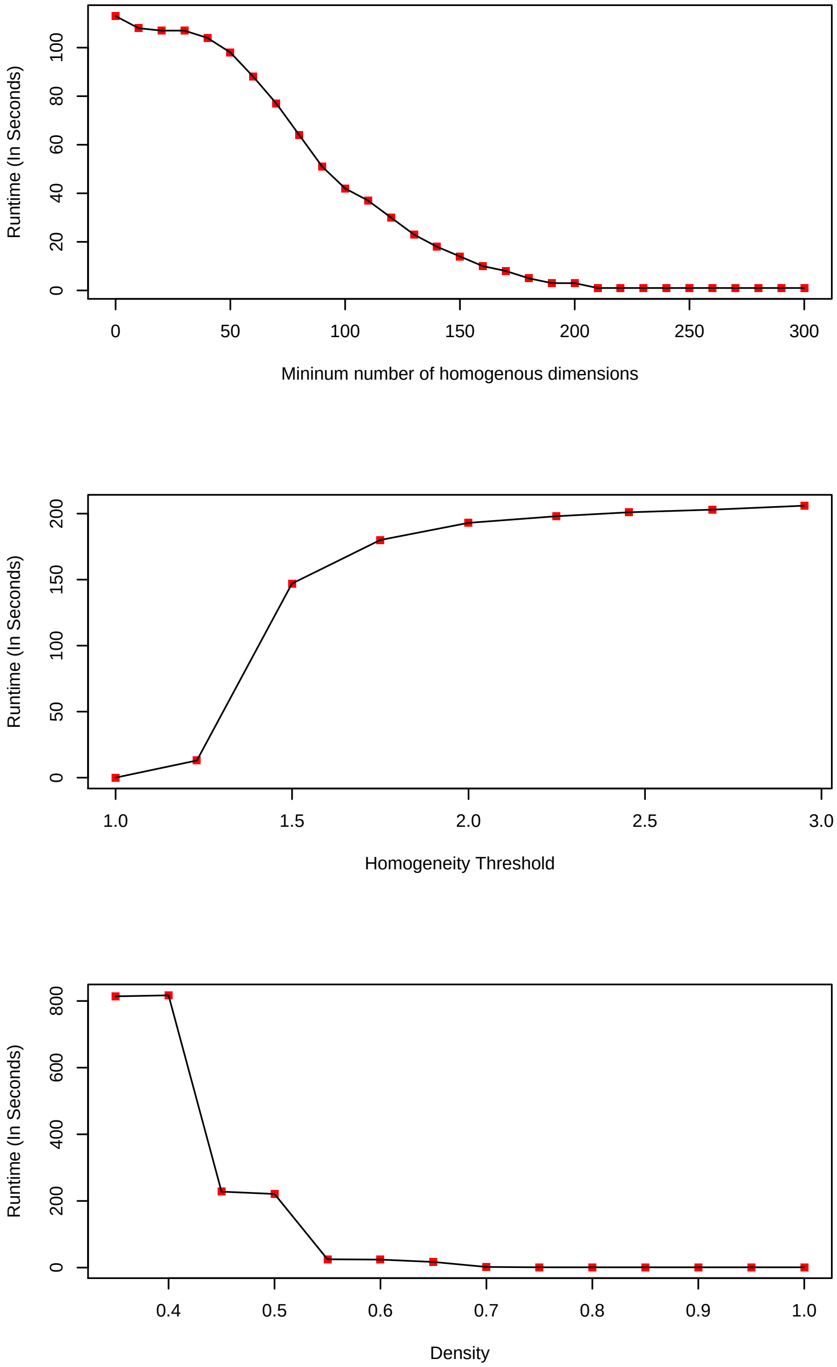

Figure 2: Runtimes on the Yeast dataset for varying dimensionality thresholds θ_d for fixed $\alpha = 0.65, \theta_{exp} = 1.25$ (top), varying co-expression thresholds θ_{exp} for fixed $\alpha = 0.65, \theta_d = 140$ where homogeneity refers to co-expression (middle) and varying density thresholds α for fixed $\theta_{exp} = 1.25, \theta_d = 140$ (bottom)

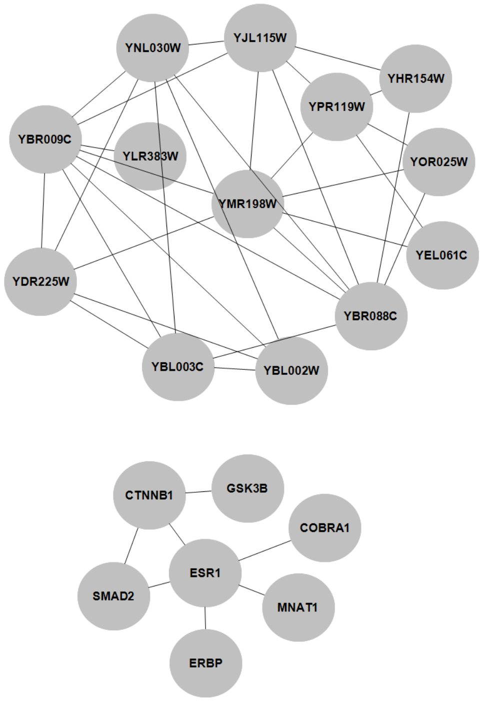

Figure 3: ~~Left~~Top: Yeast module, enriched with the GO term GO:0006333 ('Chromatin Assembly'). ~~Right~~Bottom: Human module, enriched with the GO term GO:0060070 ('Wnt Receptor Signaling Pathway through Beta-Catenin').

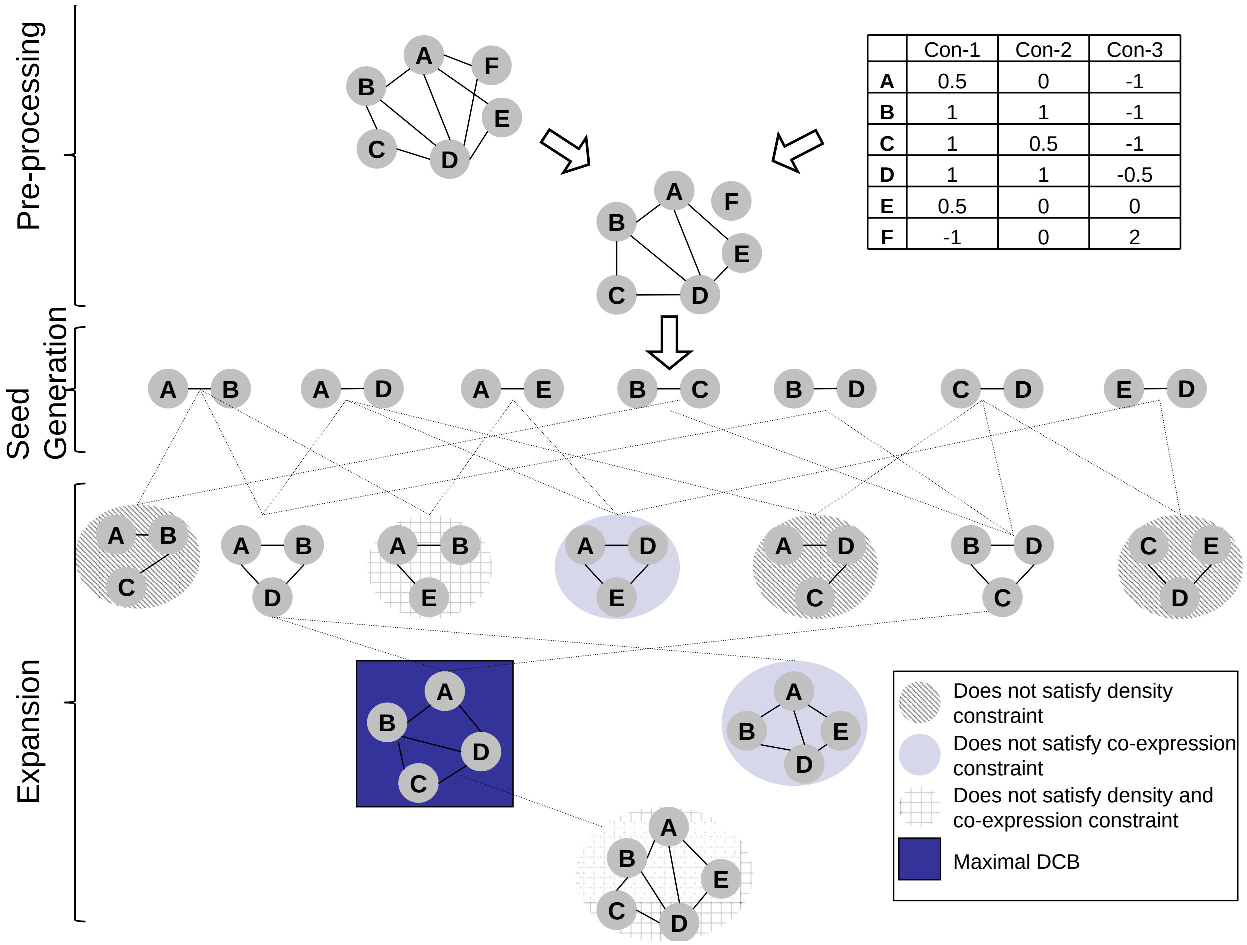

Figure 4: Illustration of the $DECOB$ algorithm on a simplified example consisting of six genes and three gene expression conditions. $DECOB$ constraints are specified by: $\alpha = 0.8$, $\theta_{exp} = 0.5$ and $\theta_d = 2$. For esthetical reasons, we have omitted B-C-D-E although, as a child of the densely connected bicluster B-C-D, it is also examined. B-C-D-E, just as A-B-D-E will be discarded since it violates the density constraint.
